# An MRI-visible nanotheranostic establishes a self-amplifying pyroptosis-STING-IFN-β circuit for CD8^+^ T cell immunoactivation

**DOI:** 10.1016/j.mtbio.2026.103338

**Published:** 2026-06-10

**Authors:** Pei Jing, Lan Wang, Xiaojuan Qiu, Liming Chen, Deyan Xie, Yongqi Yu, Bing Li, Shuang Yan, Guojun Wang, Zhirong Zhong, Wenguang Fu

**Affiliations:** a Department of Pharmacy, The Affiliated Hospital, Southwest Medical University, Luzhou, 646000, China; b School of Pharmacy, Southwest Medical University, Luzhou, 646000, China; c Department of General Surgery (Hepatobiliary Surgery), Department of Biliary-Pancreatic Center, The Affiliated Hospital, Southwest Medical University, Luzhou, 646000, China; d Metabolic Hepatobiliary and Pancreatic Diseases Key Laboratory of Luzhou City, Academician (Expert) Workstation of Sichuan Province, The Affiliated Hospital, Southwest Medical University, Luzhou, 646000, China; e Hubei Key Laboratory of Wudang Local Chinese Medicine Research, Hubei University of Medicine, Shiyan, Hubei, 442000, China; f Medical Imaging Key Laboratory of Sichuan Province, North Sichuan Medical College, Nanchong, Sichuan, 637000, China

**Keywords:** Functional nanotheranostics, Pyroptosis, cGAS-STING signaling, IFN-β, CD8^+^ T cell immunoactivation, Magnetic resonance imaging

## Abstract

Effective cancer immunotherapy relies on robust infiltration and sustained functionality of tumor-resident cytotoxic CD8^+^ T cells. Here we report a magnetic resonance imaging (MRI)-visible nanotheranostic that integrates redox modulation, inflammatory cell death, and innate immune sensitization to establish a self-amplifying pyroptosis-STING-IFN-β circuit for CD8^+^ T cell immunoactivation. The nanotheranostic is constructed through manganese ion (Mn^2+^)-catalyzed oxidative polymerization of polyphenols, enabling engineered reactive oxygen species (ROS) generation and localized metal ion availability. Functionally, intracellular ROS triggers caspase-3-dependent gasdermin E (GSDME) cleavage and pyroptosis, while concomitant mitochondrial perturbation increases cytosolic mitochondrial DNA (mtDNA) and sensitizes the cGAS-STING pathway to Mn^2+^-enhanced activation, resulting in IFN-β production. IFN-β further reinforces dendritic cells maturation and CD8^+^ T cells priming, whereas inflammatory pyroptosis amplifies mtDNA availability, together forming a self-amplifying positive-feedback circuit that magnifies antitumor immunity. *In vivo*, this coordinated mechanism promotes effective CD8^+^ T cells infiltration and memory formation. Owing to its high longitudinal relaxivity, the nanotheranostic enables clear T_1_-weighted MRI contrast with pronounced tumor accumulation and prolonged retention, supporting imaging-guided immunotherapy. This work presents a functional materials strategy that couples mechanistic immune amplification with diagnostic imaging, offering a conceptual framework for translatable immunotheranostics.

## Highlights

• Mn^2+^-doped metal-phenolic nanotheranostic with T_1_ MRI visibility
• ROS triggers caspase-3/GSDME-mediated tumor pyroptosis
• Pyroptosis-induced mtDNA release synergizes with Mn^2+^ to amplify cGAS-STING activation
• Self-amplifying pyroptosis-STING-IFN-β immune circuit
• Enhanced CD8^+^ T cell infiltration and antitumor memory formation

## Graphical abstract

**Figure:**
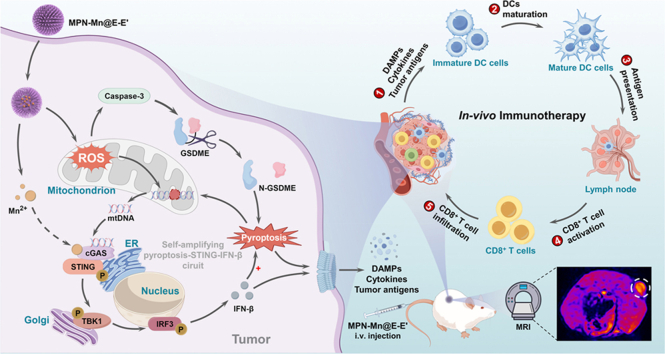

## 1 Introduction

Cytotoxic CD8^+^ T lymphocytes (CTLs) are principal effectors of antitumor immunity, eliminating malignant cells through antigen-specific recognition and cytolytic activity [1]. The magnitude and durability of immunotherapeutic efficacy critically depend on sufficient intratumoral infiltration and sustained functional competence of CD8^+^ T cells [2]. In malignant melanoma, one of the most aggressive and immunogenic skin cancers, immune checkpoint blockade has produced remarkable clinical benefit in a subset of patients [[3], [4], [5]]. Nevertheless, the overall therapeutic impact remains constrained by inefficient antigen presentation, limited T cell priming, and an immunosuppressive tumor microenvironment that restricts the recruitment and persistence of cytotoxic CD8^+^ T cells [6]. These challenges underscore the need for engineered strategies that not only increase the abundance of tumor infiltrating CD8^+^ T cells but also enhance their antigen specific functionality.

Immunogenic forms of tumor cell death have emerged as powerful initiators of antitumor immunity by bridging innate immune activation with adaptive T cell responses [7]. Among various programmed cell death (PCD) modalities, ferroptosis has recently attracted extensive attention as a reactive oxygen species (ROS)-associated cell death pathway characterized by iron-dependent lipid peroxidation and oxidative membrane damage. Owing to its strong dependence on redox dysregulation, ferroptosis-based nanomedicines have been widely developed to amplify oxidative stress and enhance antitumor efficacy [[8], [9], [10], [11], [12]]. Similar to ferroptosis, pyroptosis is another ROS-associated mode of PCD, has also attracted growing attention for its capacity to release immunostimulatory danger signals, including danger-associated molecular patterns (DAMPs), cytokines, and tumor antigens, that promote dendritic cells (DCs) maturation and antigen cross-presentation [13]. Pyroptosis is executed through proteolytic activation of gasdermin family proteins, most notably via caspase-3-dependent cleavage of gasdermin E (GSDME), which generates a pore-forming N-terminal fragment (N-GSDME) [14]. The formation of gasdermin pores leads to rapid plasma-membrane rupture and the extracellular release of danger signals. Beyond plasma membrane rupture, pyroptotic signaling can also perturb the mitochondrial membrane, resulting in the cytosolic release of mitochondrial DNA (mtDNA) as a consequence of elevated mitochondrial ROS [15,16]. These events collectively create a highly immunostimulatory milieu that favors DCs activation and subsequent CD8^+^ T cells priming, positioning pyroptosis as an effective strategy to convert immunologically “cold” tumors into inflamed, T cell-permissive lesions.

Concurrently, cytosolic DNA sensing through the cyclic GMP-AMP synthase-stimulator of interferon genes (cGAS-STING) pathway represents a central axis of innate immune surveillance in cancer [6]. mtDNA released from damaged mitochondria serves as an endogenous ligand for cGAS, driving the synthesis of the second messenger 2′3′-cyclic GMP-AMP (2′3′-cGAMP), which subsequently activates STING and initiates downstream phosphorylation cascades (e.g., TANK-binding kinase 1 (TBK1)-interferon regulatory factor 3 (IRF3), ultimately culminating in the production of type I interferons, particularly interferon-β (IFN-β), along with proinflammatory cytokines [17]. Activation of cGAS-STING in tumor cells and DCs enhances antigen presentation and co-stimulatory signaling, thus promoting robust CD8^+^ T cell responses [[18], [19], [20]]. Importantly, emerging evidence suggests that inflammatory cell death and innate immune sensing are not independent processes but can reinforce one another to amplify antitumor immunity [21,22].

Manganese (Mn) is an essential trace element with multifaceted biological functions [23]. Beyond its established functions in inducing oxidative stress through Fenton-like reactions and serving as a magnetic resonance imaging (MRI) contrast agent, Mn^2+^ has recently been identified as a potent endogenous sensitizer of nucleic acid sensing [6,24,25]. Specifically, Mn^2+^ can enhance cGAS enzymatic activity and lower the activation threshold for mtDNA detection, thereby amplifying downstream STING signaling and type I interferon responses, ultimately conferring potent CD8^+^ T cell-mediated immunomodulatory effects [6,26,27]. However, the translational application of free Mn^2+^ is limited by concerns regarding systemic accumulation and metal-associated toxicity [28]. Metal-phenolic networks (MPNs) offer a versatile materials platform to overcome these limitations. The coordination between phenolic ligands and metal ions enables the rapid construction of tunable supramolecular networks capable of self-assembling into nanoparticles, featuring facile preparation, structural versatility, and preferential accumulation within tumor tissues [29,30]. Importantly, the pH responsive properties of many MPNs allow for nanostructure disassembly and controlled metal ions release in the acidic tumor microenvironment [30]. Epigallocatechin-3-gallate (EGCG), a representative natural polyphenol and the major bioactive constituent of green tea, contains vicinal tri-phenolic groups in its molecular structure, which endow it with strong metal-chelating capability to form stable metal-phenolic assemblies [31,32]. In addition, EGCG has been shown to induce ROS generation in tumor cells, leading to DNA damage and the initiation of pyroptotic cell death, thereby activating antitumor immune responses and promoting CD8^+^ T cell activation [33,34]. Notably, Mn^2+^ can undergo a Fenton-like reaction with endogenous H_2_O_2_ in tumor cells to generate highly reactive hydroxyl radicals, thereby synergizing with EGCG, which induces intracellular ROS production and H_2_O_2_ accumulation, to further amplify oxidative stress.

**Scheme 1:**
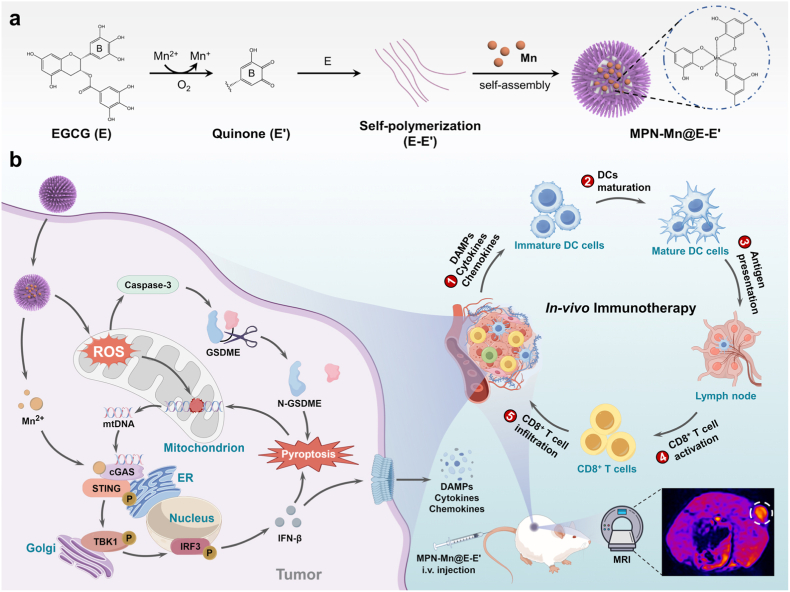
Schematic illustration of the MRI-visible nanotheranostic-mediated immunoactivation mechanism. The nanotheranostic induces intracellular ROS generation, leading to caspase-3-dependent GSDME cleavage and pyroptotic tumor cell death, which releases DAMPs and tumor antigens to promote DCs maturation and antigen presentation. Concurrently, ROS induced mitochondrial perturbation increases cytosolic mtDNA, while localized Mn^2+^ availability sensitizes the cGAS-STING pathway, resulting in IFN-β production. IFN-β further reinforces DCs activation and CD8^+^ T cells priming, whereas inflammatory pyroptosis amplifies mtDNA availability, together forming a closed, self-amplifying pyroptosis-STING-IFN-β positive-feedback circuit that potentiates antitumor immunity. In parallel, the intrinsic MRI visibility of the nanotheranostic enables noninvasive visualization of tumor accumulation to support imaging-guided immunotherapy.

Building on these insights, we engineered a manganese-doped metal-phenolic nanomedicine (MPN-Mn@E-E′) that integrates redox perturbation, inflammatory cell death, innate immune sensitization, and noninvasive imaging into a unified immunoactivation cascade (Scheme 1). Following cellular internalization, MPN-Mn@E-E′ induces robust intracellular ROS generation, which activates caspase-3-dependent cleavage of GSDME and triggers pyroptotic tumor cell death. This inflammatory death program promotes DCs maturation and antigen presentation, initiating cytotoxic CD8^+^ T cells responses. In parallel, ROS induced mitochondrial damage facilitates the cytosolic release of mtDNA, while the localized liberation of Mn^2+^ from the metal-phenolic framework sensitizes cGAS to mtDNA and amplifies downstream cGAS-STING signaling, leading to enhanced IFN-β production. IFN-β further reinforces DCs activation and CD8^+^ T cells priming, intensifying antitumor immune responses. Notably, the combined action of inflammatory pyroptosis and interferon signaling increases mitochondrial stress and mtDNA availability, thereby feeding back to strengthen cGAS-STING activation and establishing a self-amplifying pyroptosis-STING-IFN-β positive feedback circuit**.** Moreover, the manganese-based framework confers intrinsic MRI contrast capability, enabling noninvasive visualization of tumor accumulation and supporting imaging-guided translational deployment. Together, this work delineates a rational materials engineering strategy to remodel the tumor immune microenvironment and potentiate CD8^+^ T cell-mediated antitumor immunity through a mechanistically defined closed-loop circuit.

## 2 Results and discussion

### 2.1 Synthesis and characterization of MPN-Mn@E-E′

**Fig. 1:**
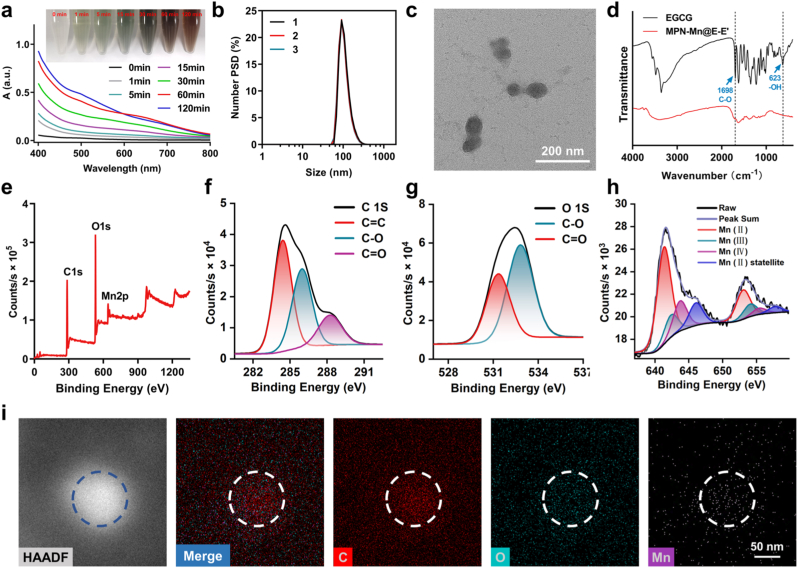
Structural and physicochemical characterization of MPN-Mn@E-E'. (a) UV-vis absorption spectra recorded at different time points during the formation of MPN-Mn@E-E'; inset shows corresponding color changes of the reaction solution. (b) DLS analysis and (c) TEM images of MPN-Mn@E-E'. (d) FTIR spectra of EGCG and MPN-Mn@E-E'. XPS analysis including (e) full survey spectrum and (f) high-resolution spectra of C 1s, (g) O 1s, and (h) Mn 2p. (i) Elemental mapping images confirming the uniform distribution of constituent elements within MPN-Mn@E-E′ nanoparticles.

The manganese-doped metal-phenolic nanoparticles MPN-Mn@E-E′ were fabricated via Mn^2+^-catalyzed oxidative polymerization of EGCG in N-2-hydroxyethylpiperazine-N′-2-ethanesulfonic acid (HEPES) buffer (Scheme 1a). The nanoparticles obtained under the G4 condition showed optimal performance (Fig. S1). Progressive solution darkening accompanied by the emergence of broad ultraviolet-visible (UV-vis) absorption across 400-700 nm confirmed successful oxidative polymerization of EGCG (Fig. 1a). High-performance liquid chromatography (HPLC) further verified the gradual consumption of free EGCG during nanoparticle formation (Fig. S2).

Dynamic light scattering (DLS) measurement revealed hydrodynamic diameters below 120 nm with narrow size distributions, while transmission electron microscopy (TEM) images showed well-defined spherical morphologies (Fig. 1b and c, Fig. S3 and S4), a size regime favorable for passive tumor accumulation. The MPN-Mn@E-E′ nanoparticles exhibited a negative surface potential (−35.7 mV) (Fig. S4), indicative of good colloidal stability and reduced nonspecific interactions under physiological conditions.

Fourier-transform infrared (FTIR) spectra demonstrated attenuation of characteristic phenolic O-H stretching vibrations, suggesting coordination between Mn^2+^ and EGCG hydroxyl groups (Fig. 1d). X-ray photoelectron spectroscopy (XPS) further confirmed Mn incorporation and oxidative transformation of EGCG, as evidenced by increased carbonyl content in the C 1s and O 1s spectra and the dominance of Mn^2+^ species in the Mn 2p region (Fig. 1e–h and Fig. S5). Elemental mapping showed homogeneous Mn distribution throughout the nanostructures (Fig. 1i), and inductively coupled plasma mass spectrometry (ICP-MS) analysis quantified a Mn content of 8.86 wt% (Fig. S6). The nanoparticles maintained stable hydrodynamic sizes in various physiological media for at least 72 h, underscoring MPN-Mn@E-E′ exhibited excellent colloidal robustness (Fig. S7).

To verify the pH-responsive sensitivity of MPN-Mn@E-E′, the release behavior of Mn^2+^ in solutions at 37 °C with pH values of 7.4, 6.5, and 5.5 was evaluated using the equilibrium dialysis method, followed by quantification via ICP-MS. As shown in Fig. S8, the release of Mn^2+^ from MPN-Mn@E-E′ was markedly slower under physiological conditions (pH 7.4), with a cumulative release of only 8.73% after 72 h, indicating that MPN-Mn@E-E′ can effectively minimize Mn^2+^ leakage during storage and systemic blood circulation. In contrast, Mn^2+^ release was substantially accelerated under acidic conditions, particularly at pH 5.5, where the cumulative release increased to 66.07%. This phenomenon is mainly attributed to protonation-induced disruption of the coordination interactions, which subsequently promotes the dissociation and release of Mn^2+^. As these conditions mimic the acidic tumor microenvironment, the pH-dependent release behavior demonstrates the suitability of MPN-Mn@E-E′ for tumor-targeted therapy.

### 2.2 In vitro induction of pyroptosis and activation of the cGAS-STING pathway

Successful cellular internalization is a prerequisite for nanoparticles to exert their biological activities and therapeutic functions. Therefore, we first evaluated the cellular internalization capability of the nanoparticles by quantifying the intracellular Mn content in B16F10 cells using ICP-MS. As exhibited in Fig. S9, the intracellular Mn content in B16F10 cells was markedly increased following nanoparticle treatment and gradually accumulated over time, indicating that the nanoparticles could be efficiently internalized by the cells. EGCG has been reported to induce intracellular ROS generation in tumor cells, while Mn^2+^, as a redox-active transition metal, can further amplify oxidative stress through Fenton-like reactions [24,33]. Accordingly, intracellular ROS levels were assessed using 2′,7′-dichlorodihydrofluorescein diacetate (DCFH-DA) following treatment with MPN-Mn@E-E'. As shown in Fig. S10a, confocal laser scanning microscopy (CLSM) revealed a marked elevation of intracellular ROS in B16F10 cells after incubation with MnCl_2_, EGCG, and MPN-Mn@E-E′, with the most pronounced fluorescence signal observed in the MPN-Mn@E-E′ group. Consistent with these observations, flow cytometry analysis demonstrated that the ROS-positive population in the MPN-Mn@E-E′ group reached 37.53%, corresponding to a 2.81- and 1.32-fold increase relative to the MnCl_2_ and EGCG groups, respectively (Fig. S10b and c).

**Fig. 2:**
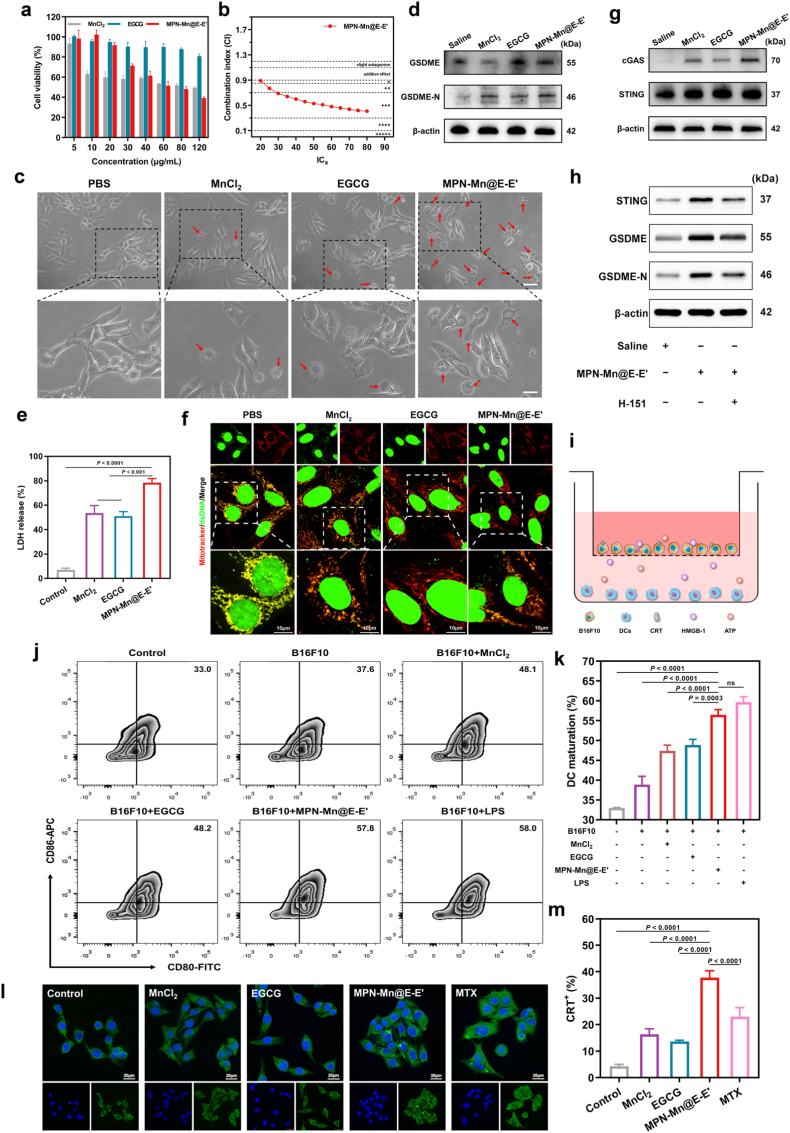
*In vitro* induction of pyroptosis and DCs maturation by MPN-Mn@E-E'. (a) Cell viability of B16F10 cells treated with MnCl_2_, EGCG, and MPN-Mn@E-E′ for 48 h. (b) Combination index analysis of MPN-Mn@E-E′ against B16F10 cells indicating synergistic effects. The degree of synergism was categorized into five levels: slight (+), moderate (++), synergism (+++), strong synergism (++++), and very strong synergism (+++++). Representative microscopy images of (c) pyroptotic morphology and (d) western blot detecting the expression of GSDME and its cleaved form (GSDME-N) in B16F10 cells under various treatments. (e) LDH release of B16F10 cells in the supernatant. (f) CLSM images indicated the release of damaged mtDNA from the mitochondria. Green: dsDNA, Red: mitochondria. (g) Western blot assessing the expression levels of cGAS and STING. (h) Effects of the combined treatment with MPN-Mn@E-E′ and the STING inhibitor H-151 on the expression of related proteins. (i) Schematic diagram depicting the setup of the transwell coculture system. (j) Representative flow cytometry plots and (k) quantitative analysis of DCs maturation (CD11c^+^CD80^+^CD86^+^) following stimulation with LPS or MPN-Mn@E-E'. (l) Confocal laser scanning microscopy images showing CRT exposure (green) in B16F10 tumor cells following various treatments. (m) Flow cytometric quantification of CRT exposure in B16F10 tumor cells following various treatments. Data are presented as mean ± SD and analyzed by one-way ANOVA with Bonferroni post hoc test.

The robust ROS generating capability of MPN-Mn@E-E′ translated into enhanced cytotoxicity against B16F10 cells. As shown in Fig. 2a, after 48 h incubation, free MnCl_2_ exhibited moderate cytotoxicity with an IC_50_ of 113.42 μg/mL, whereas EGCG alone showed only marginal antitumor activity (IC_50_ > 120 μg/mL). Notably, assembly into the MPN-Mn@E-E′ nanoparticles significantly potentiated cytotoxic efficacy, yielding an IC_50_ of 90.02 μg/mL, representing at least a 1.56- and 1.26-fold enhancement compared with EGCG and MnCl_2_, respectively. These results indicate that MPN-Mn@E-E′ effectively reduces the required drug dosage while maintaining antitumor efficacy, suggesting a synergistic therapeutic effect. This synergy was further quantified using the cooperativity index (CI) [35]. As shown in Fig. 2b, CI values ranging from 0.41 to 0.89 were obtained for MPN-Mn@E-E′, confirming strong synergistic anticancer activity. Consistently, calcein acetoxymethyl (AM)/propidium iodide (PI) live/dead staining revealed a substantially higher proportion of dead cells in the MPN-Mn@E-E′ group compared with the MnCl_2_ and EGCG groups, further corroborating its superior cytotoxicity (Fig. S11).

Excessive ROS is a well-recognized molecular trigger of pyroptosis, a form of programmed cell death characterized by prominent cell swelling and plasma membrane rupture [36]. As shown in Fig. 2c, B16F10 cells treated with MPN-Mn@E-E′ exhibited pronounced membrane swelling (indicated by red arrows), indicative of pyroptotic morphology. ROS-induced caspase-3-dependent cleavage and activation of GSDME drives pyroptosis, wherein the cleaved N-terminal GSDME mediates membrane pore formation [36,37]. Accordingly, the expression levels of caspase-3, GSDME and N-GSDME were examined. As shown in Fig. 2d, Figs. S12 and S13, all three proteins were most abundantly expressed in the MPN-Mn@E-E′ group, providing direct molecular evidence that MPN-Mn@E-E′ effectively induces GSDME-mediated pyroptosis. In addition, lactate dehydrogenase (LDH)and interleukin-18 (IL-18) play critical roles in pyroptosis. We then measured the extracellular LDH and IL-18 levels in the culture supernatants. Fig. 2e and Fig. S14 shows that the LDH concentration and IL-18 expression levels were markedly elevated in B16F10 cells treated with MPN-Mn@E-E′ compared with those in all other groups, indicating that MPN-Mn@E-E′ potentiates pyroptosis in an efficient manner.

Excessive oxidative stress can further induce mitochondrial dysfunction. Therefore, we next investigated the effects of MPN-Mn@E-E′ on mitochondria in B16F10 cells. The JC-1 probe was employed to evaluate the mitochondrial membrane potential (ΔΨm). Healthy mitochondria maintain a high ΔΨm, which facilitates the accumulation of JC-1 aggregates within the mitochondrial matrix and generates red fluorescence. In contrast, depolarized mitochondria with reduced membrane potential retain JC-1 in its monomeric form, resulting in green fluorescence emission. As shown in Fig. S15, the PBS group exhibited intense red fluorescence, whereas the MPN-Mn@E-E′ group displayed prominent green fluorescence, indicating a substantial decrease in mitochondrial membrane potential. The loss of ΔΨm further suggested increased mitochondrial membrane permeability. Given that excessive ROS generation and pyroptotic signaling can induce severe mtDNA damage, we further evaluated the release of mtDNA from mitochondria. A green fluorescent dsDNA probe was used to label mtDNA, while mitochondria were stained with the red mitochondrial tracker dye (Mitotracker). As expected, obvious fluorescence colocalization was observed in the PBS group, indicating that mtDNA was predominantly retained within mitochondria. In contrast, the MPN-Mn@E-E′ group exhibited markedly weakened green fluorescence within mitochondria, and portions of the mtDNA-associated green fluorescence were dispersed outside the mitochondria, suggesting the release of mtDNA into the cytoplasm (Fig. 2f). The ROS- and pyroptosis-associated mtDNA release, together with Mn^2+^ sensitization, synergistically activates the cGAS-STING pathway [6,38]. We therefore investigated whether MPN-Mn@E-E′ could activate this pathway in B16F10 cells. As shown in Fig. S16, MPN-Mn@E-E′ induced the most pronounced membrane rupture at 24 h post-treatment. Western blot analysis further revealed that, compared with MnCl_2_ or EGCG alone, MPN-Mn@E-E′ elicited the strongest upregulation of cGAS and STING expression (Fig. 2g and Fig. S17). This enhanced activation is likely attributable to pyroptosis-associated mtDNA release, which synergistically sensitizes the Mn^2+^-cGAS-STING signaling axis.

**Fig. 3:**
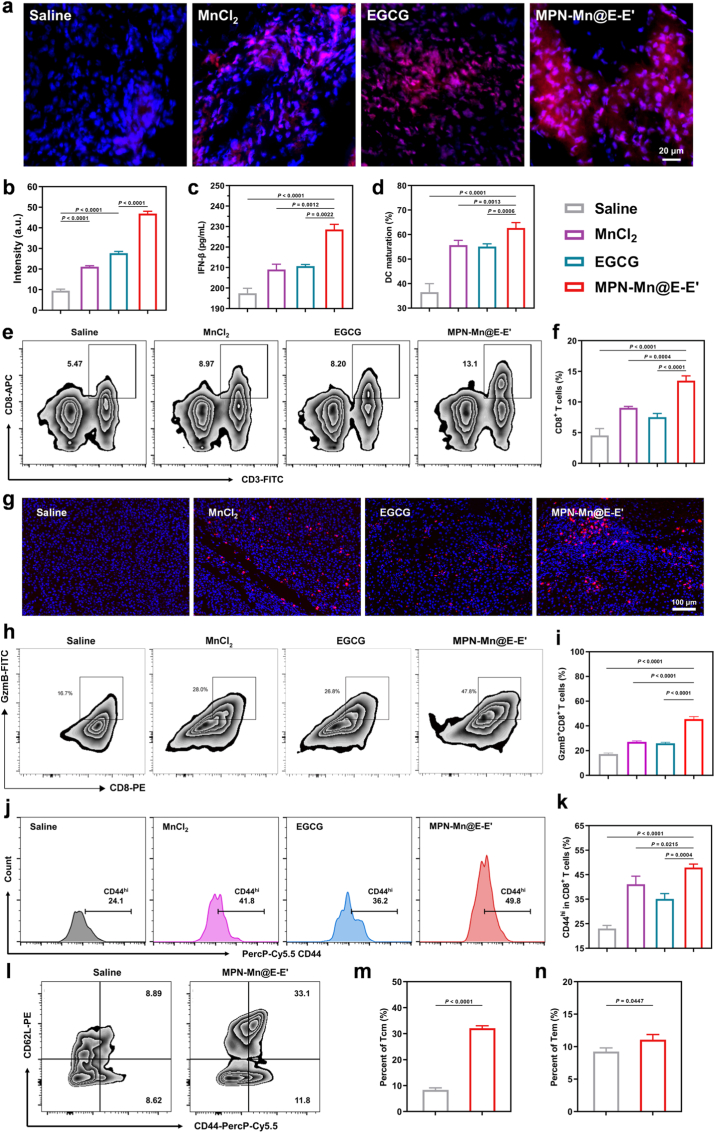
*In vivo* activation of CD8^+^ T cells-mediated immune responses by MPN-Mn@E-E'. (a) Immunofluorescence staining showing ROS generation (red) in tumor tissues after various treatments. (b) Fluorescence quantitative analysis of the ROS generation in tumor tissues after various treatments. (c) Serum IFN-β levels quantified by enzyme-linked immunosorbent assay (ELISA) kits. (d) Maturation status of splenic DCs assessed by flow cytometry. (e) Representative flow cytometry plots and (f) quantitative analysis of CD8^+^ T cells gated from CD3^+^ T cells in tumors. (g) Immunofluorescence staining of CD8^+^ T cells (red) in tumor tissues after various treatments. (h) Representative flow cytometry plots and (i) quantitative analysis of GzmB^+^ cells after gating on CD8^+^ T cells in tumors. (j) Representative flow cytometry plots and (k) quantitative analysis of CD44^hi^ cells after gating on CD8^+^ T cells in tumors. (l) Representative flow cytometry plots and quantitative analysis of (m) central memory T cells (T_CM_) and (n) effector memory T cells (T_EM_) (CD44^+^CD62L^+^) gated from CD3^+^CD8^+^ T cells in spleens. All quantitative data are shown as mean ± SD. Statistical significance was determined by one-way ANOVA with Bonferroni post hoc test.

Pyroptosis and STING activation can stimulate the secretion of the proinflammatory cytokine IFN-β, while IFN-β further reinforces inflammatory pyroptosis and amplifies mtDNA availability, thereby further activating the STING pathway and ultimately forming a self-amplifying positive-feedback pyroptosis-STING-IFN-β circuit. To verify the existence of this circuit, we specifically disrupted the STING pathway using the inhibitor H-151. As shown in Fig. 2h, Figs. S18 and S19, consistent with the results shown in Fig. 2d and g, Fig. 3c, treatment with MPN-Mn@E-E′ markedly increased the expression levels of STING, GSDME, and GSDME-N proteins in B16F10 cells, accompanied by enhanced secretion of IFN-β. In contrast, co-treatment with MPN-Mn@E-E′ and the STING pathway inhibitor H-151 not only markedly reduced STING expression, but also simultaneously decreased the protein levels of GSDME and GSDME-N, as well as IFN-β secretion. These findings indicate that blockade of STING attenuates the expression of downstream pyroptosis-related proteins and the production of the proinflammatory cytokine IFN-β, thereby providing additional evidence supporting the existence of a positive-feedback pyroptosis-STING-IFN-β circuit.

Given that pyroptosis-STING-IFN-β circuit plays a central role in antitumor immunity by promoting DCs maturation, we next evaluated the immunostimulatory capacity of MPN-Mn@E-E'. An *in vitro* transwell co-culture system of B16F10 cells and DCs was established (Fig. 2i), and DCs maturation was quantified by flow cytometric analysis of CD11c^+^CD80^+^CD86^+^ cells. As shown in Fig. 2j and k, relative to untreated controls, MnCl_2_ and EGCG increased DCs maturation by 1.16- and 1.18-fold, respectively, whereas MPN-Mn@E− E′ elevated the proportion of mature DCs to 1.36-fold (*p* < 0.0001), a level comparable to that induced by the lipopolysaccharide (LPS) positive control. These results demonstrate that MPN-Mn@E-E′ effectively promotes DCs maturation and exhibits strong potential to initiate antitumor immune responses.

In addition to STING activation, pyroptosis is known to induce immunogenic cell death (ICD), thereby further enhancing DCs maturation [39]. To evaluate the ICD-inducing capability of MPN-Mn@E-E′, calreticulin (CRT) exposure, high-mobility group box 1 (HMGB-1) and adenosine triphosphate (ATP) release, were assessed as hallmark ICD markers. CLSM imaging and flow cytometry analyses revealed that CRT translocation to the cell surface was significantly higher in the MPN-Mn@E-E′ group than in the MnCl_2_ and EGCG groups, and even exceeded that of the methotrexate (MTX) positive control (Fig. 2l and m and Fig. S20). Consistently, HMGB-1 and ATP release followed a similar trend (Fig. S21, S22 and S23), further confirming the potet ICD-inducing capacity of MPN-Mn@E-E'.

### 2.3 In vivo activation of CD8^+^ T cells

Metal-associated toxicity is a major concern that restricts the biomedical application of metal-based biomaterials. Although manganese is an essential trace element, excessive Mn^2+^ exposure may induce systemic toxicity [28,40]. Therefore, prior to *in vivo* studies, the biosafety of MPN-Mn@E-E′ was systematically assessed. An *in vitro* hemolysis assay was performed to evaluate the hemocompatibility of MPN-Mn@E-E'. As shown in Fig. S24, the nanoparticles exhibited negligible hemolytic activity (hemolysis rate <5%) even at concentrations as high as 320 μg/mL. Subsequently, BALB/c mice were intravenously administered saline, MnCl_2_, EGCG, and MPN-Mn@E-E′ every two days for a total of seven injections. Fourteen days after the final administration, major organs, including the heart, liver, spleen, lung, and kidney, were harvested for histopathological analysis. Hematoxylin and eosin (H&E) staining revealed no discernible tissue damage or necrosis in any treatment group (Fig. S25). Consistently, serum biochemical indices, including alanine aminotransferase (ALT), aspartate aminotransferase (AST), alkaline phosphatase (ALP), urea (UREA), and creatinine (CREA), showed no significant differences among the groups (Fig. S26). Notably, no obvious toxicity was observed even in the MnCl_2_-treated mice, indicating that incorporation of an equivalent Mn dose into MPN-Mn@E-E′ does not induce additional metal-associated toxicity. Collectively, these results demonstrate the favorable *in vivo* biosafety profile of MPN-Mn@E-E'.

Cytotoxic T lymphocytes, primarily CD8^+^ T cells, play a central role in antitumor immunity by recognizing tumor-associated antigens and executing tumor cell killing. activation of CD8^+^ T cells critically depends on efficient antigen presentation by mature DCs. Accordingly, we next investigated whether MPN-Mn@E-E′ could promote DCs maturation and subsequent CD8^+^ T cells activation *in vivo*. B16F10 tumor-bearing BALB/c mice were intravenously treated with saline, MnCl_2_, EGCG, and MPN-Mn@E-E'. Dihydroethidium (DHE) staining of tumor sections revealed that the MPN-Mn@E-E′ group exhibited the strongest ROS signal, which was 4.94-fold higher than that of the saline group (*p* < 0.0001), indicating potent intratumoral ROS generation (Fig. 3a and b). This elevated oxidative stress is expected to robustly induce tumor cell pyroptosis and promote the release of mtDNA, thereby sensitizing the Mn^2+^-cGAS-STING signaling axis. Consistent with this mechanism, MPN-Mn@E-E′ treatment elicited the most pronounced secretion of inflammatory cytokines associated with STING activation, including IFN-β and IL-12. As shown in Fig. 3c and Fig. S27, the levels of IFN-β and IL-12 were increased by 1.12- and 1.23-fold, respectively, compared with the saline group (*p* < 0.001). These cytokines not only directly facilitate DCs maturation but also further exacerbate tumor cell pyroptosis, thereby amplifying immunogenic cell death. In line with this notion, immunofluorescence staining revealed the highest levels of CRT exposure and HMGB-1 release in tumors treated with MPN-Mn@E-E′, confirming its strong ICD-inducing capability (Fig. S28 and Fig. S29). We next evaluated DCs maturation in the spleens of tumor-bearing mice. Flow cytometric analysis demonstrated that MPN-Mn@E-E′ markedly increased the proportion of mature DCs to 65.66%, corresponding to 1.45-, 1.13-, and 1.14-fold increases compared with saline, MnCl_2_, and EGCG treatments, respectively (Fig. 3d and Fig. S30). Given that mature DCs are essential for antigen presentation and T cells priming, we further examined CD8^+^ T cells activation and infiltration. Flow cytometry revealed that the frequency of intratumoral CD8^+^ T cells in the MPN-Mn@E-E′ group was 2.96-, 1.49-, and 1.79-fold higher than those in the saline, MnCl_2_, and EGCG groups, respectively (Fig. 3e and f). These findings were further corroborated by immunofluorescence staining, which showed the highest density of CD8^+^ T cells infiltration within tumor tissues following MPN-Mn@E-E′ treatment (Fig. 3g). In addition to evaluating CD8^+^ T cells infiltration, we further assessed the expression levels of Granzyme B (GzmB) and IFN-γ to verify the effect of MPN-Mn@E-E′ on the functionality of CD8^+^ T cells. As shown in Fig. 3h and i, the number of GzmB^+^CD8^+^ T cells in tumor tissues was highest in the MPN-Mn@E-E′ group compared with all other groups. Moreover, the secretion level of the pro-inflammatory cytokine IFN-γ in the serum of mice treated with MPN-Mn@E-E′ was also markedly higher than that in the other treatment groups (Fig. S31). The elevated levels of GzmB and IFN-γ further demonstrated that MPN-Mn@E-E′ effectively enhanced the functionality of CD8^+^ T cells.

To further assess the functional status of activated CD8^+^ T cells, the expression of CD44, a hallmark marker of effector and memory cytotoxic T cells, was analyzed. As expected, MPN-Mn@E-E′ treatment significantly increased the proportion of CD44^hi^CD8^+^ T cells within the tumor microenvironment compared with all other treatment groups (Fig. 3j and k), indicating enhanced cytotoxic potential. Finally, the induction of long-term antitumor immune memory was evaluated by quantifying splenic central memory T cells (T_CM_) and effector memory T cells (T_EM_) [41]. As shown in Fig. 3l-n, 17 days after treatment cessation, both T_CM_ and T_EM_ populations were significantly elevated in the MPN-Mn@E-E′ group relative to the saline control (*p* < 0.01), with T_CM_ exhibiting the most pronounced increase (*p* < 0.0001). These results indicate that MPN-Mn@E-E′-mediated tumor-catalyzed immunotherapy not only elicits robust effector immune responses but also establishes durable immune memory, which may contribute to the long-term suppression of tumor recurrence and metastasis.

### 2.4 Immunotheranostic efficacy of MPN-Mn@E-E′

**Fig. 4:**
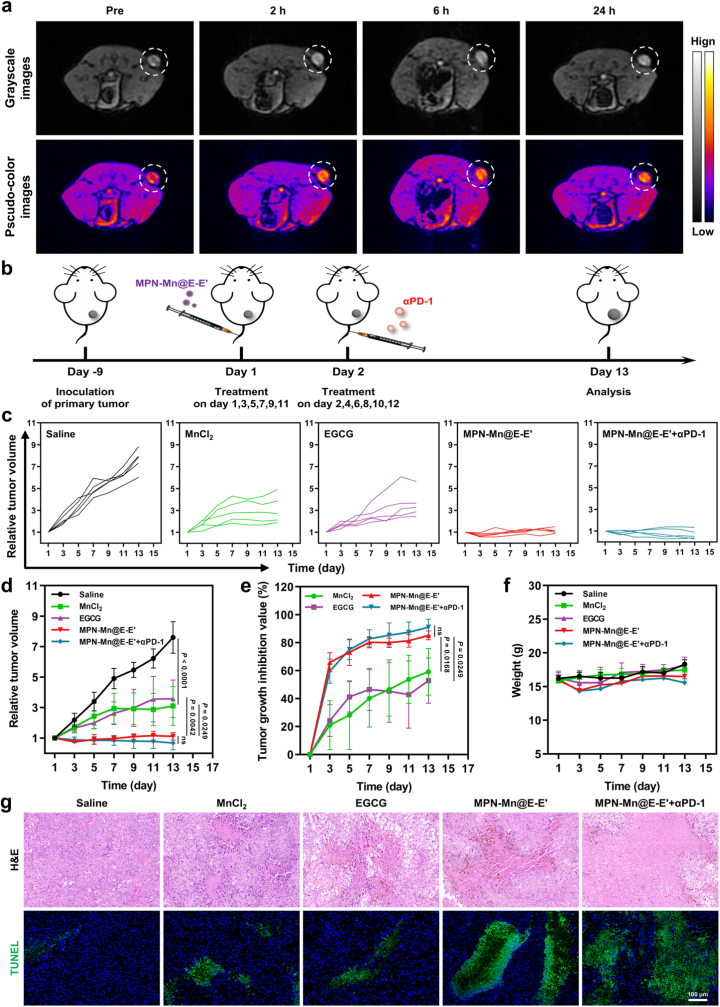
*In vivo* theranostic performance of MPN-Mn@E-E′ in B16F10 tumor-bearing mice. (a) T_1_-weighted MR images of mice at various time points following intravenous injection of MPN-Mn@E-E'. (b) Schematic illustration of the *in vivo* treatment protocol. (c) Individual tumor growth curves and (d) average relative tumor volume changes following different treatments. (e) Tumor growth inhibition rates for each treatment group. (f) Body weight curves of mice over the treatment period. (g) Representative images of H&E and TUNEL staining in post-treatment tumor sections. Values are reported as mean ± SD and were analyzed via one-way ANOVA coupled with Bonferroni post hoc testing.

Nanoplatforms constructed based on EGCG-derived carriers are mainly distributed in the liver and kidneys, which serve as their major metabolic and clearance pathways [31,42]. Since the immunotherapeutic efficacy of nanoplatforms is closely associated with their distribution within tumor tissues, we further evaluated the intratumoral accumulation of MPN-Mn@E-E′ in the B16F10 tumor tissue. Manganese possesses a high magnetic spin moment, rapid water exchange kinetics, and favorable biocompatibility, rendering Mn^2+^-chelated or coordination-based nanomaterials attractive candidates for T_1_-weighted MRI. In line with this rationale, the longitudinal relaxivity (r_1_) of MPN-Mn@E-E′, a key parameter governing T_1_ contrast efficiency, was systematically evaluated. As shown in Fig. S32, MPN-Mn@E-E′ exhibited a high r_1_ value of 40.18 mM^−1^ s^−1^, which is comparable to or even higher than those of manganese-based contrast agents recently reported in the literature [[43], [44], [45]]. For instance, Liu et al. reported a manganese-based nanocomposite RMnMels, with an r_1_ value of 2.49 mM^−1^ s^−1^ [44]; while Sun et al. developed a Mn(II)-chelated ionic covalent organic framework nanoparticle Mn-iCOF exhibiting an r_1_ value of 8.02 mM^−1^ s^−1^ [45]. Moreover, MPN-Mn@E-E′ also demonstrated a clear advantage over the clinically established gold-standard contrast agent Gd-DOTA (r_1_ = 3.87 mM^−1^ s^−1^) [45]. Based on the high r_1_ relaxivity, the *in vivo* T_1_-weighted MRI performance was further evaluated in B16F10 tumor-bearing BALB/c mice using a 3.0 T MRI scanner. As shown in Fig. 4a, a pronounced enhancement of T_1_-weighted signals was observed at the tumor sites following intravenous administration, indicating preferential tumor accumulation of MPN-Mn@E-E'. Semi-quantitative analysis revealed that the maximal signal enhancement was achieved at 6 h post-injection, while a discernible MRI signal remained detectable even at 24 h (Fig. S33). Collectively, these results demonstrate that the excellent relaxivity and imaging performance of MPN-Mn@E-E′ enable sensitive tumor visualization and effective monitoring of its accumulation and retention in tumors, which is closely associated with its therapeutic potential and ultimately contributes to improved diagnosis and treatment of melanoma.

The *in vivo* antitumor efficacy of MPN-Mn@E-E′ was subsequently evaluated using a B16F10 melanoma model. Mice were intravenously administered saline, MnCl_2_, EGCG, and MPN-Mn@E-E′ according to the treatment schedule illustrated in Fig. 4b, with tumor volume and body weight monitored every two days. As shown in Fig. 4c and d, tumors in the saline-treated group grew rapidly, whereas moderate tumor growth inhibition was observed in the MnCl_2_ and EGCG groups. In stark contrast, tumors in the MPN-Mn@E-E′-treated group exhibited minimal growth throughout the treatment period. Quantitative analysis revealed that MPN-Mn@E-E′ achieved a tumor growth inhibition rate of 85.29%, which is 1.44- and 1.61-fold higher than those of the MnCl_2_ and EGCG groups, respectively (*p* < 0.01) (Fig. 4e).

Notably, co-administration of the immune checkpoint inhibitor αPD-1 did not result in a statistically significant further enhancement of antitumor efficacy compared with MPN-Mn@E-E′ treatment alone (*p* > 0.05), suggesting that MPN-Mn@E-E′ elicits potent intrinsic antitumor activity without requiring additional checkpoint blockade. Throughout the treatment period, no significant body weight loss was observed in any group, indicating favorable *in vivo* tolerability of MPN-Mn@E-E' (Fig. 4f). Upon completion of treatment, tumors from representative mice in each group were harvested for histopathological evaluation (Fig. S34) [[46], [47], [48]]. Consistent with the tumor growth inhibition results, H&E staining and TdT-mediated dUTP nick end labeling (TUNEL) analysis revealed extensive tumor cell necrosis and apoptosis in tumors treated with MPN-Mn@E-E′ and MPN-Mn@E-E' + αPD-1, with no appreciable difference between the two groups (Fig. 4g).

### 2.5 In vivo CD8^+^ T cell depletion test

**Fig. 5:**
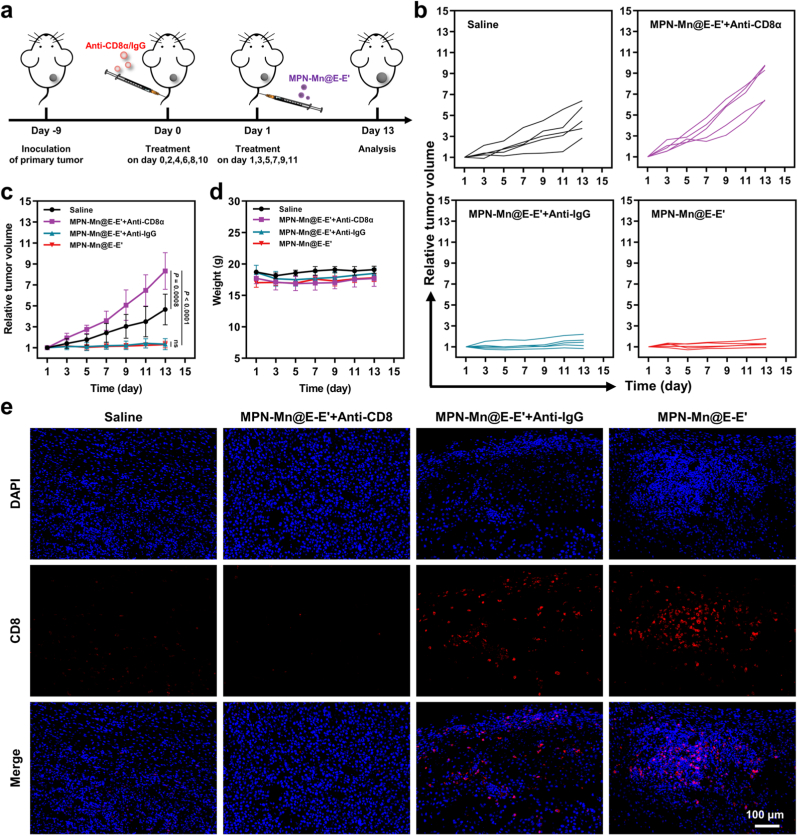
CD8^+^ T cell depletion confirms the CD8^+^ T cell-dependent antitumor efficacy of MPN-Mn@E-E'. (a) Schematic illustration of the CD8^+^ T cell depletion protocol in tumor-bearing mice. (b) Individual and (c) average relative tumor volume changes following different treatments. (d) Body weight changes during the treatment period. (e) Representative immunofluorescence staining of tumor-infiltrating CD8^+^ T cells in tumor tissues after treatment. Data are presented as mean ± SD; significance was determined by one-way ANOVA followed by Bonferroni correction.

CD8^+^ T cells, also referred to as cytotoxic T lymphocytes, are the principal effector cells responsible for direct tumor cell elimination in antitumor immune responses. As demonstrated in Fig. 3, MPN-Mn@E-E′ effectively promotes CD8^+^ T cells activation *in vivo*. To further determine whether the antitumor efficacy of MPN-Mn@E-E′ is causally dependent on CD8^+^ T cells-mediated immunity, a CD8^+^ T cells depletion study was performed in B16F10 tumor-bearing mice using a CD8α-depleting antibody, with an isotype-matched IgG antibody serving as the control (Fig. 5a). Mice were subsequently treated via tail vein injection with saline, MPN-Mn@E-E′, MPN-Mn@E-E' + anti-CD8α, and MPN-Mn@E-E' + IgG, and tumor growth was continuously monitored.

As shown in Fig. 5b and c, tumors in the saline-treated group grew progressively throughout the treatment period. Notably, depletion of CD8^+^ T cells markedly abrogated the therapeutic efficacy of MPN-Mn@E-E′, as evidenced by significantly accelerated tumor growth in the MPN-Mn@E-E' + anti-CD8α group. At the end of the treatment, the final tumor volume in this group reached 1.79-fold that of the saline group (p = 0.0008). In contrast, robust tumor growth suppression was maintained in both the MPN-Mn@E-E′ and MPN-Mn@E-E' + IgG groups, indicating that the antitumor efficacy of MPN-Mn@E-E′ critically depends on the presence of functional CD8^+^ T cells. Throughout the treatment course, mice in all groups exhibited a modest increase in body weight, further confirming the absence of appreciable systemic toxicity associated with MPN-Mn@E-E′ administration (Fig. 4d).

To further corroborate the effects of CD8^+^ T cells depletion at the tissue level, tumors were harvested after completion of treatment and subjected to CD8 immunofluorescence staining. As shown in Fig. S35 and F ig. 5e, CD8^+^ T cells were markedly depleted in tumors from the MPN-Mn@E-E' + anti-CD8α group, whereas pronounced infiltration and activation of CD8^+^ T cells were detected in tumors treated with MPN-Mn@E-E′ alone or in combination with IgG. Collectively, these results provide direct evidence that CD8^+^ T cells are indispensable for the antitumor efficacy of MPN-Mn@E-E′ and firmly establish CD8^+^ T cells-mediated immune responses as the dominant mechanism underlying its therapeutic activity.

Taken together, these results establish MPN-Mn@E-E′ as a tumor-catalyzed immunotheranostic nanoplatform that integrates oxidative stress amplification, pyroptosis induction, and innate immune activation to elicit robust antitumor immunity. By amplifying intratumoral ROS, MPN-Mn@E-E′ triggers GSDME-dependent pyroptosis and immunogenic cell death, leading to the release of tumor-derived danger signals and mtDNA. In parallel, Mn^2+^ sensitizes the cGAS-STING pathway, resulting in amplified inflammatory cytokine production and efficient DCs maturation. This coordinated activation of innate immunity subsequently promotes potent CD8^+^ T cells infiltration, functional activation, and long-term immune memory, which collectively mediate durable tumor suppression *in vivo*. Importantly, CD8^+^ T cells depletion abrogates the therapeutic efficacy of MPN-Mn@E-E′, providing direct causal evidence that its antitumor activity is predominantly immune-mediated. Together with its favorable biosafety profile and T_1_-weighted MRI capability, this work highlights a rational strategy for converting tumor cells into endogenous immune activators and underscores the potential of pyroptosis-STING-CD8^+^ T cells axis driven nanomedicine for effective cancer immunotheranostics.

## 3 Conclusion

In conclusion, this work presents an imaging enabled metal-phenolic nanoplatform that integrates materials engineering with immuno-mechanistic design to amplify antitumor immunity. Distinct from many previously reported metal-polyphenol nanoplatforms that rely on multicomponent formulations or structurally complex assemblies, the present nanoparticle is constructed solely from EGCG and manganese through a simple coordination-driven assembly process, featuring a structurally concise composition, well-defined pharmacological components, and mechanistically clear bioactivity. By coupling ROS driven, caspase-3-dependent GSDME-mediated pyroptosis with Mn^2+^-sensitized cGAS-STING activation, the system establishes a self-amplifying pyroptosis-STING-IFN-β positive feedback circuit that promotes DCs maturation and robust CD8^+^ T cells priming. The coordinated reinforcement between inflammatory cell death and innate immune sensing underlies the enhanced magnitude and functionality of tumor infiltrating CD8^+^ T cells. Beyond immune activation, the incorporation of manganese confers intrinsic T_1_-weighted MRI contrast capability, enabling noninvasive visualization of tumor accumulation and providing a translationally relevant imaging readout. Therefore, this nanoplatform not only achieves synergistic immunomodulation through a mechanistically defined immune amplification loop, but also integrates diagnostic imaging and therapeutic functionality within a single minimalistic system. By unifying redox perturbation, inflammatory cell death, innate immune sensitization, and imaging guidance within a single platform, this work establishes a generalizable strategy for designing functional immunoactive materials. The mechanistically defined closed-loop circuit described here offers a conceptual blueprint for imaging-assisted precision immunotherapy and may inform the development of next-generation nanomaterials for controllable and durable cancer immune modulation.

## 4 Materials and methods

### 4.1 Materials

Manganese chloride (MnCl_2_), N-2-hydroxyethylpiperazine-N′-2-ethanesulfonic acid (HEPES), and potassium bromide (KBr) were purchased from Aladdin (Shanghai, China). Epigallocatechin gallate-3-gallate (EGCG, 98%), dimethyl sulfoxide (DMSO, analytical grade), methotrexate (MTX), methanol (≥99.9%), acetonitrile (≥99.9%), 3-(4,5-dimethylthiazol-2-yl)-2,5-diphenyltetrazolium bromide (MTT, ≥99.9%), and glacial acetic acid (≥99.9%) were supplied by Macklin Co., Ltd. (Shanghai, China). 5% bovine serum albumin (BSA) blocking solution and 2′,7′-dichlorodihydrofluorescein diacetate (DCFH-DA) were acquired from Beijing Solarbio Science & Technology Co., Ltd. (Beijing, China). Trypsin and lipopolysaccharide (LPS) were procured from Beyotime Biotechnology (Shanghai, China) and Invitrogen (USA), respectively. Antibodies were sourced from the following suppliers: Proteintech (Wuhan, China) supplied those against cyclic GMP-AMP synthase (cGAS), stimulator of interferon genes (STING), gasdermin E (GSDME) and its pore-forming N-terminal fragment (N-GSDME), as well as the conjugated antibodies allophycocyanin (APC)/Cyanine 7 (Cy7) anti-mouse CD3, phycoerythrin (PE) anti-mouse CD11c, PE anti-mouse CD62L, fluorescein isothiocyanate (FITC) anti-mouse CD80, and APC anti-mouse CD86. BD Biosciences (USA) was the source of FITC anti-mouse CD3 and APC anti-mouse CD8α antibodies. Peridinin-chlorophyll-protein (PerCP)-Cyanine5.5 (Cy5.5) anti-mouse CD44 antibodies were obtained from BioLegend, Inc. (San Diego, CA, USA). Finally, antibodies targeting programmed cell death protein 1 (PD-1) and anti-mouse CD8, along with rat IgG2b antibodies, were purchased from Bio X Cell (Lebanon, NH, USA).

### 4.2 Cells

The B16F10 cell line was procured from the School of Pharmacy, Southwest Medical University, and was maintained in Dulbecco's modified Eagle's medium (DMEM; Servicebio, Wuhan, China) containing 10% Fetal bovine serum (FBS; Servicebio, Wuhan, China) and 1% penicillin-streptomycin (50 U/mL each), under standard culture conditions (37 °C, 5% CO_2_, humidified atmosphere). Primary bone marrow-derived dendritic cells (BMDCs), harvested from mice, were grown in 1640 medium supplemented with 10% FBS, 1% penicillin-streptomycin, granulocyte-macrophage colony-stimulating factor (GM-CSF, 20 ng/mL), and interleukin-4 (IL-4, 10 ng/mL) under the same incubation conditions.

### 4.3 Animals

BALB/c mice (6-8 weeks old) were acquired from Beijing Huafukang Biotechnology Co., Ltd. (Beijing, China) and housed under specific pathogen-free (SPF) conditions with free access to food and water. All experimental procedures involving animals were reviewed and approved by the Institutional Animal Care and Use Committee (IACUC) of Southwest Medical University, and were performed in strict compliance with the national guidelines for animal experimentation in China.

### 4.4 Optimization of preparation conditions

Adapted from a previously established method [49], MPN-Mn@E-E′ nanoparticles were synthesized under vigorous stirring in a thermostated water bath maintained at 25 °C. Specifically, 2 mM manganese chloride (MnCl_2_) were combined with 2.5 mM EGCG in 10 mM N-2-hydroxyethylpiperazine-N′-2-ethanesulfonic acid (HEPES) buffer (pH 7.4). The resultant nanoparticles were then harvested after a 1-h reaction via centrifugation (16 000 rpm, 20 min) and underwent two washing cycles with fresh HEPES buffer (10 mM, pH 7.4). Single-factor optimization experiments were conducted by varying Mn^2+^ concentration, solution pH, stirring speed, and reaction temperature to investigate their effects on nanoparticle formation. The reaction conditions were systematically evaluated based on the hydrodynamic size of the resulting nanoparticles, and the optimal preparation parameters were determined accordingly.

### 4.5 Synthesis and characterization of MPN-Mn@E-E′

The preparation of MPN-Mn@E-E′ involved mixing MnCl_2_ (25 mM) and EGCG (2.5 mM) in HEPES buffer (10 mM, pH 8.0) at 37 °C with stirring. Post-reaction (60 min), nanoparticles were centrifuged (25 000 g, 10 min), washed with HEPES buffer (pH 7.4), and stored at 4 °C. An ultraviolet-visible (UV-vis) spectrophotometer was used to dynamically monitor absorbance.

Dynamic light scattering (DLS) measurements provided hydrodynamic size and ζ-potential at 25 °C. Transmission electron microscopy (TEM) imaging revealed morphology. Fourier-transform infrared (FTIR) spectroscopy identified functional groups. X-ray photoelectron spectroscopy (XPS) analysis determined elemental composition/chemical states. Inductively coupled plasma mass spectrometry (ICP-MS) quantified manganese. Stability was tested over 72 h in different media (HEPES, water, saline, culture medium) by tracking size changes.

### 4.6 Cytotoxicity assessment

The *in vitro* cytotoxicity of MnCl_2_, EGCG, and MPN-Mn@E-E′ was evaluated in B16F10 cells using the 3-(4,5-dimethylthiazol-2-yl)-2,5-diphenyltetrazolium bromide (MTT) assay. Cells were plated in 96-well plates at 8 × 10^3^ cells/well. Following adhesion, the medium was changed to fresh DMEM containing 10% FBS and supplemented with various concentrations of MnCl_2_, EGCG, and MPN-Mn@E-E' (0, 5, 10, 20, 30, 40, 80, and 120 μg/mL). After 48 h of treatment, cell viability was assessed via the MTT assay, and the results were analyzed with CompuSyn 2.0 software to assess the comparative antitumor efficacy of MPN-Mn@E-E′ relative to the corresponding physical mixtures.

Live/dead staining was performed as follows: B16F10 cells were cultured overnight in confocal dishes at 3 × 10^5^ cells/well (37 °C). Cells were then exposed to varying concentrations of MPN-Mn@E-E′, MnCl_2_, and EGCG for 24 h; the concentrations of MnCl_2_ and EGCG matched those contained in the nanoparticles. After treatment, cells were washed and subjected to staining with calceinn B16F10 c AM and propidium iodide (PI) for 30 min. Finally, fluorescence was visualized using a confocal laser scanning microscope (CLSM).

### 4.7 Intracellular reactive oxygen species (ROS) levels *in vitro*

Intracellular ROS generation iells was evaluated using the fluorescent probe 2′,7′-dichlorodihydrofluorescein diacetate (DCFH-DA), with quantification performed by both confocal imaging and flow cytometry. For confocal imaging, cells were respectively seeded in confocal dishes and 24-well plates at densities of 3 × 10^5^ and 1 × 10^5^ cells per well overnight. Cells were then exposed to complete medium containing MnCl_2_ ([Mn] = 2.8 μg/mL), EGCG (37.2 μg/mL), and MPN-Mn@E-E' (40 μg/mL) for 12 h. Following this treatment, cells were loaded with 5 μM DCFH-DA for 30 min, washed with Phosphate-buffered saline (PBS), and imaged by CLSM.

For flow cytometric quantification, under the same treatment conditions, cells were detached by trypsin, washed, and similarly incubated with 5 μM DCFH-DA for 30 min. After three additional PBS washes, the fluorescence intensity, indicative of ROS levels, was measured using a flow cytometer.

### 4.8 Lactate dehydrogenase (LDH) and interleukin-18 (IL-18) release assays

B16F10 cells were seeded into 96-well plates at a density of 5 × 10^3^ cells per well and incubated for 24 h. Subsequently, the cells were co-incubated with MnCl_2_ ([Mn] = 2.8 μg/mL), EGCG (37.2 μg/mL), and MPN-Mn@E-E' (40 μg/mL) at 37 °C for 12 h. The culture medium was then collected and centrifuged at 400 g for 5 min. The supernatants from each well were harvested and analyzed using an LDH cytotoxicity assay kit and a mouse IL-18 ELISA kit, respectively.

### 4.9 Mitochondrial damage and mtDNA release assay

Mitochondrial membrane potential was assessed using the JC-1 probe. B16F10 cells were seeded in confocal culture dishes and incubated with MnCl_2_ ([Mn] = 2.8 μg/mL), EGCG (37.2 μg/mL), and MPN-Mn@E-E' (40 μg/mL) for 12 h. Subsequently, the cells were stained with the JC-1 probe for 30 min to label mitochondria, washed with PBS, and observed by confocal laser scanning microscopy (CLSM).

Following the above procedure, B16F10 cells were seeded in confocal culture dishes and incubated with MnCl_2_ ([Mn] = 2.8 μg/mL), EGCG (37.2 μg/mL), and MPN-Mn@E-E' (40 μg/mL) for 12 h. The cells were then stained with a dsDNA probe and mitochondrial tracker dye (Mitotracker) for confocal imaging.

### 4.10 Immunogenic cell death (ICD) detection

Immunofluorescence (IF) and flow cytometric analyses for CRT exposure, HMGB-1 and ATP release were performed on B16F10 cells treated with MPN-Mn@E-E' (40 μg/mL, 24 h). The IF protocols for calreticulin (CRT) exposure and high-mobility group box 1 (HMGB-1) were identical except for the primary antibody: after fixation (4% paraformaldehyde (PFA), 5 min) and permeabilization (0.1% Triton X-100, 30 min), cells were blocked with 5% BSA and incubated overnight at 4 °C with either anti-CRT or anti-HMGB-1 primary antibody. Following washing, cells were stained with CoraLite488-conjugated (for CRT) or CoraLite594-conjugated (for HMGB-1) secondary antibodies for 2 h, counterstained with 4′,6-diamidino-2-phenylindole (DAPI), and imaged by CLSM.

For flow cytometric analysis of CRT (a similar protocol was followed): Treated cells were harvested, fixed (1% PFA, 5 min), washed, and blocked (5% BSA, 37 °C, 30 min). Subsequent incubations were with anti-CRT primary antibody (2 h) and CoraLite488-conjugated secondary antibody (1.5 h, dark). After final washes, cells were resuspended in PBS for flow cytometry analysis.

For ATP detection, the supernatants of B16F10 cells subjected to different treatments were collected and centrifuged at 1500 rpm for 5 min, followed by determination of ATP concentration using an ATP assay kit. An HMGB-1 assay kit was similarly used to evaluate HMGB-1 release, following the same experimental procedures as those used for the ATP assay.

### 4.11 In vitro dendritic cells activation

To assess dendritic cells (DCs) maturation, BMDCs were isolated from BALB/c mice for use in a transwell coculture system, following an established protocol. In this setup, B16F10 cells subjected to various treatments were placed in the upper chamber, while BMDCs occupied the lower chamber. After a 24-h coculture period, DCs were collected and subjected to surface staining with fluorescently labeled antibodies against mouse CD11c (PE), CD80 (FITC), and CD86 (APC). This was followed by PBS washing and subsequent analysis via flow cytometry.

### 4.12 Hemolysis assay

The hemocompatibility of the materials was evaluated using a hemolysis assay. Briefly, fresh EDTA-anticoagulated whole blood was collected from the retro-orbital venous plexus of BALB/c mice and centrifuged at 1000 rpm for 10 min. Subsequently, 0.9 mL of MPN-Mn@E-E′ solutions at different concentrations (20, 40, 80, 160, and 320 μg/mL) was mixed with 0.1 mL of 10% (v/v) red blood cell suspension. After incubation at room temperature for 2 h, the samples were centrifuged at 1500 rpm for 10 min, and the supernatants were collected and photographed. Finally, the absorbance of hemoglobin in each sample was measured at 541 nm using a UV-vis spectrophotometer, and the hemolysis rate was calculated accordingly.

### 4.13 Dendritic cell maturation and immune T cell analysis

A subcutaneous B16F10 tumor model was established by inoculating 5 × 10^6^ cells into the right flank of female BALB/c mice. When tumor volumes reached ∼100 mm^3^, mice received intravenous MPN-Mn@E-E' (40.0 mg/kg) via the tail vein every two days for a total of three doses. Mice were euthanized 48 h after the final injection, and blood, tumors, and spleens were harvested for subsequent analyses.

Quantification of serum interferon-β (IFN-β) and IL-12 was performed using commercial enzyme-linked immunosorbent assay (ELISA) kits. For immunofluorescence, tumors were sectioned and stained for CRT and HMGB-1 according to standard protocols. Spleens were processed into single-cell suspensions via mechanical dissociation and filtration through a 70 μm strainer. For analysis of DCs maturation, splenocytes were blocked with 5% BSA and immunostained with fluorescent antibodies against CD11c (PE), CD80 (FITC), and CD86 (APC) prior to flow cytometry.

To assess *in vivo* CD8^+^ T cell activation, single-cell suspensions derived from tumor tissues were labeled with antibodies against CD3 (FITC), CD8 (APC), and CD44 (PerCP-Cy5.5) for flow cytometric analysis.

### 4.14 In vivo antitumor effeciency

A subcutaneous tumor model was established by inoculating 1 × 10^6^ B16F10 cells into the right flank of 6- to 8-week-old BALB/c mice. When tumor volumes approached 100 mm^3^, mice were randomized into five treatment cohorts (n = 5 per group): (1) saline, (2) MnCl_2_, (3) EGCG, (4) MPN-Mn@E-E′, and (5) MPN-Mn@E-E' + αPD-1. All treatments were administered intravenously via the tail vein and dosed according to body weight.

The saline group received vehicle only, while groups (4) and (5) were treated with MPN-Mn@E-E′ at a dose of 40 mg/kg (based on nanoparticle content). All treatments were administered intravenously every two days for a total of six doses. Specifically for group (5), the αPD-1 antibody (1 mg/kg) was co-administered, with the first dose given on the second treatment day and subsequent doses following the same two-day interval. Tumor size and body weight were measured every other day. Tumor volume was estimated according to the formula:

(1) V = a × b^2^ / 2

Where a and b correspond tot he longest and shortest diameters, respectively.

### 4.15 Analysis of memory T cells in the spleen

To assess the induction of immune memory, a separate tumor rechallenge model was established. BALB/c mice received subcutaneous inoculations of 1 × 10^6^ B16F10 cells into the right flank. When the average tumor volume reached approximately 100 mm ^3^, mice were randomly allocated into two cohorts (n = 5): (1) saline control and (2) MPN-Mn@E-E′ treatment. Administered on an every-other-day basis, the treatment group received six intravenous doses of MPN-Mn@E-E' (40 mg/kg), whereas control mice were administered an equivalent volume of saline following the identical schedule.

On day 29 post-inoculation, spleens were harvested and dissociated into single-cell suspensions via mechanical disruption and filtration through a 70 μm strainer. For memory CD8^+^ T cell phenotyping, splenocytes were labeled with PE-conjugated anti-CD62L and PerCP-Cy5.5-conjugated anti-CD44 antibodies, followed by flow cytometric analysis.

### 4.16 CD8^+^ T cell depletion study

Tumor-bearing mouse models were established by subcutaneous injection of 1 × 10^6^ B16F10 cells into BALB/c mice. Upon reaching a volume of approximately 100 mm^3^, tumor-bearing mice were randomized into four experimental groups (n = 5 per group): (1) saline, (2) MPN-Mn@E-E′, (3) MPN-Mn@E-E' + anti-CD8, and (4) MPN-Mn@E-E' + isotype IgG control. Mice in all groups were treated with MPN-Mn@E-E′ via tail vein injection on a schedule of one dose every two days, for a total of six administrations.

For CD8^+^ T cell depletion, anti-CD8 antibody or isotype control antibody (1 mg/kg) was administered intravenously 24 h prior to nanoparticle treatment in groups (3) and (4), respectively. Tumor growth was monitored by measuring tumor size every other day.

### 4.17 Statistical analysis

All data were reported as mean ± standard deviation (SD). The number of biological replicates is specified in the corresponding experimental section. Statistical analyses were performed using GraphPad Prism, with p < 0.05 considered statistically significant.

## CRediT authorship contribution statement

**Pei Jing:** Conceptualization, Investigation, Methodology, Validation, Writing – original draft. **Lan Wang:** Formal analysis, Methodology, Validation. **Xiaojuan Qiu:** Data curation, Formal analysis, Methodology. **Liming Chen:** Formal analysis, Methodology, Validation. **Deyan Xie:** Data curation, Validation. **Yongqi Yu:** Formal analysis, Investigation. **Bing Li:** Data curation. **Shuang Yan:** Investigation. **Guojun Wang:** Conceptualization, Resources, Supervision. **Zhirong Zhong:** Funding acquisition, Resources, Supervision. **Wenguang Fu:** Funding acquisition, Supervision, Writing – review & editing.

## Declaration of competing interest

The authors declare that they have no known competing financial interests or personal relationships that could have appeared to influence the work reported in this paper.

## Data Availability

Data will be made available on request.
