# Supplementary material for: An MRI-visible nanotheranostic establishes a self-amplifying pyroptosis-STING-IFN-β circuit for CD8+ T cell immunoactivation
Source: Mater Today Bio. 2026 Jun 10;39:103338. doi: 10.1016/j.mtbio.2026.103338 (PMC13277642; doi:10.1016/j.mtbio.2026.103338)
Supplement: Multimedia component 1 [file mmc1.pdf]

Supporting Information for

An MRI-visible nanotheranostic establishes a self-amplifying pyroptosis-STING-IFN-β circuit for $CD8^+$ T cell immunoactivation

Pei Jing[a,f,1], Lan Wang[b,1], Xiaojuan Qiu[c,d,1], Liming Chen[b,1], Deyan Xie[b], Yongqi Yu[b], Bing Li[e], Shuang Yan[f], Guojun Wang[a,*], Zhirong Zhong[b,*], Wenguang Fu[c,d,*]

[a]*Department of Pharmacy, The Affiliated Hospital, Southwest Medical University, Luzhou 646000, China*
[b]*School of Pharmacy, Southwest Medical University, Luzhou 646000, China*
[c]*Department of General Surgery (Hepatobiliary Surgery), Department of Biliary-Pancreatic Center, The Affiliated Hospital, Southwest Medical University, Luzhou 646000, China*
[d]*Metabolic Hepatobiliary and Pancreatic Diseases Key Laboratory of Luzhou City, Academician (Expert) Workstation of Sichuan Province, The Affiliated Hospital, Southwest Medical University, Luzhou 646000, China*
[e]*Hubei Key Laboratory of Wudang Local Chinese Medicine Research, Hubei University of Medicine, Shiyan 442000, Hubei, China*
[f]*Medical Imaging Key Laboratory of Sichuan Province, North Sichuan Medical College, Nanchong 637000, Sichuan, China*

[1] These authors contributed equally to this work.

* Corresponding authors: wanggj7532@163.com; zhongzhirong@126.com; fuwg@swmu.edu.cn

a

Group	EGCG	$MnCl_2$	pH	Speed (rpm)	Temperature (℃)
G1	5000 μL, 2.5 mM	200 μL, 25 mM	7.4	600	37
G2	5000 μL, 2.5 mM	200 μL, 25 mM	7.6	600	37
G3	5000 μL, 2.5 mM	200 μL, 25 mM	7.8	600	37
G4	5000 μL, 2.5 mM	200 μL, 25 mM	8.0	600	37
G5	5000 μL, 2.5 mM	200 μL, 25 mM	8.2	600	37
G6	5000 μL, 2.5 mM	200 μL, 25 mM	8.0	300	37
G7	5000 μL, 2.5 mM	200 μL, 25 mM	8.0	900	37
G8	5000 μL, 2.5 mM	200 μL, 25 mM	8.0	600	25
G9	5000 μL, 2.5 mM	100 μL, 25 mM	8.0	600	37
G10	5000 μL, 2.5 mM	400 μL, 25 mM	8.0	600	37

b

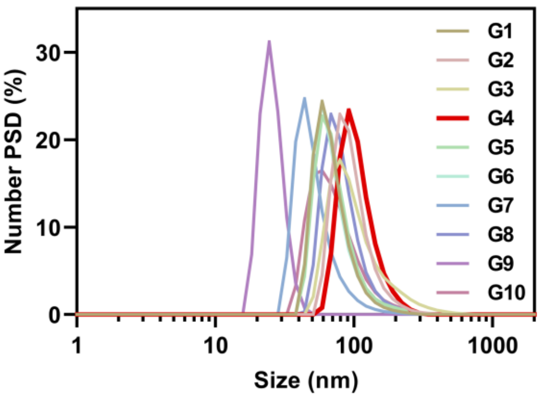

Fig. S1. Optimization of preparation conditions for MPN-Mn@E-E'. (a) Schematic illustration of experimental grouping for condition optimization. (b) Particle size distributions of MPN-Mn@E-E' obtained under different reaction conditions.

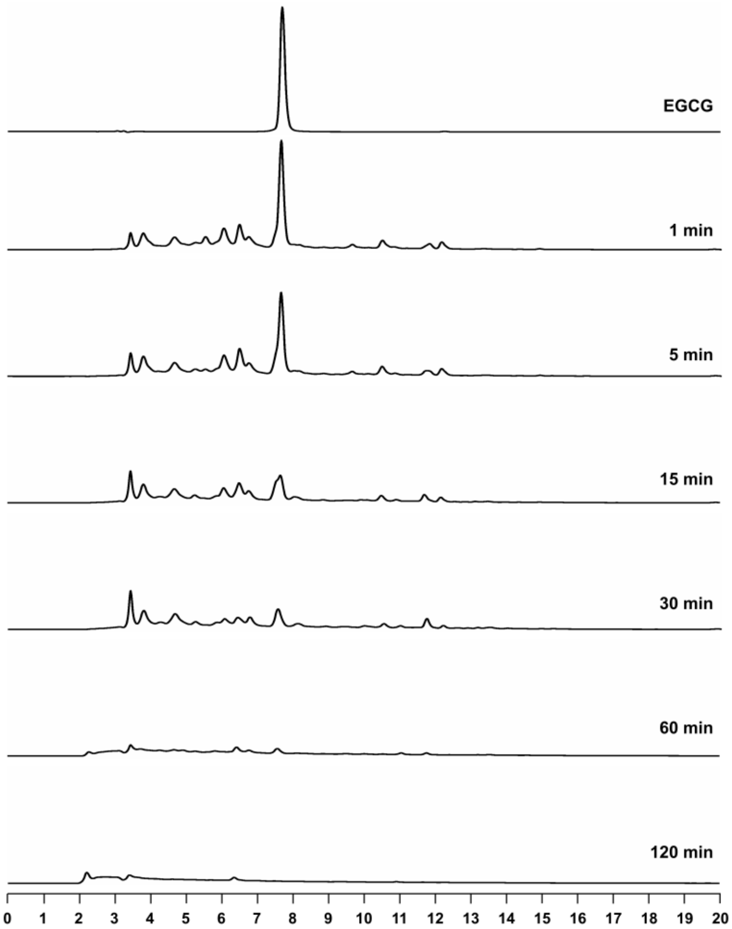

Fig. S2. Reaction kinetics during the formation of MPN-Mn@E-E'. High-performance liquid chromatography (HPLC) chromatograms of nanoparticle reaction mixtures collected at different time points during synthesis.

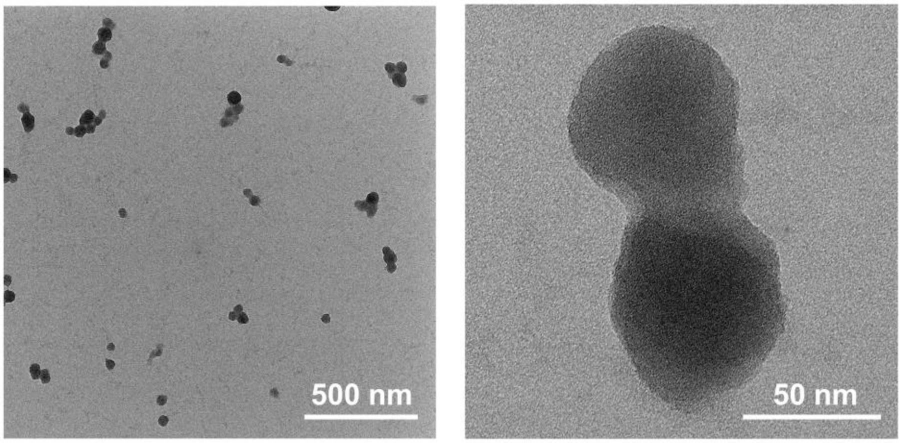

Fig. S3. Morphological characterization of MPN-Mn@E-E'. Representative transmission electron microscopy (TEM) images of MPN-Mn@E-E' at different magnifications.

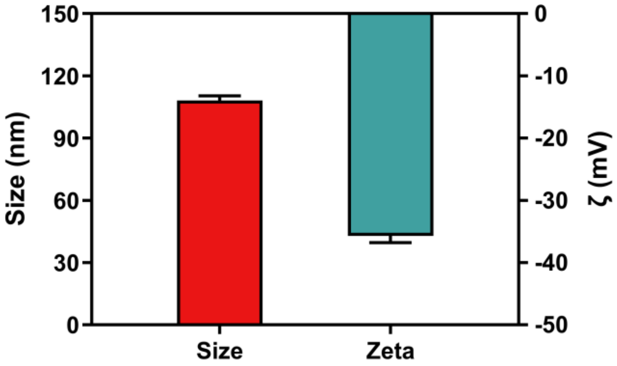

Fig. S4. Colloidal properties of MPN-Mn@E-E'. Average hydrodynamic diameter and zeta potential of MPN-Mn@E-E' measured by dynamic light scattering (DLS).

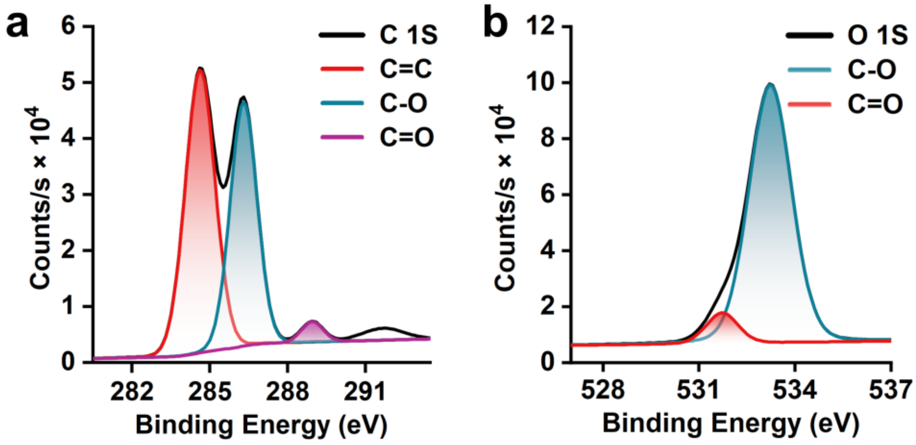

Fig. S5. X-ray photoelectron spectroscopy (XPS) characterization of EGCG. XPS spectra of (a) C 1s and (b) O 1s regions of EGCG.

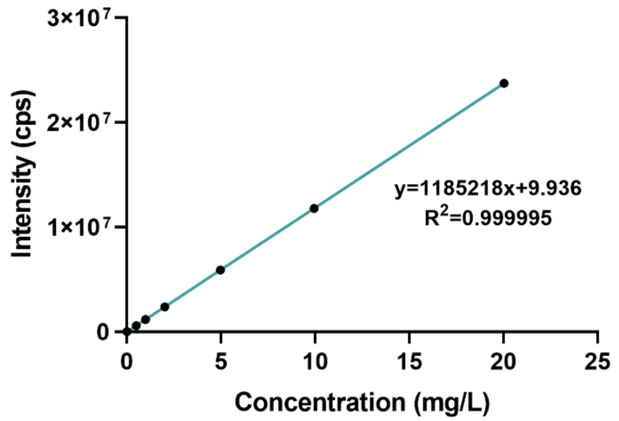

Fig. S6. Quantification of manganese content. Calibration curve for manganese concentration determined by inductively coupled plasma mass spectrometry (ICP-MS).

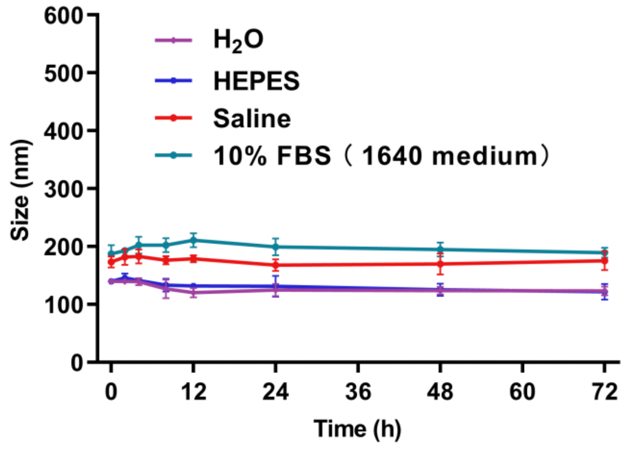

Fig. S7. Colloidal stability of MPN-Mn@E-E'. Stability evaluation of MPN-Mn@E-E' in different liquid environments over time.

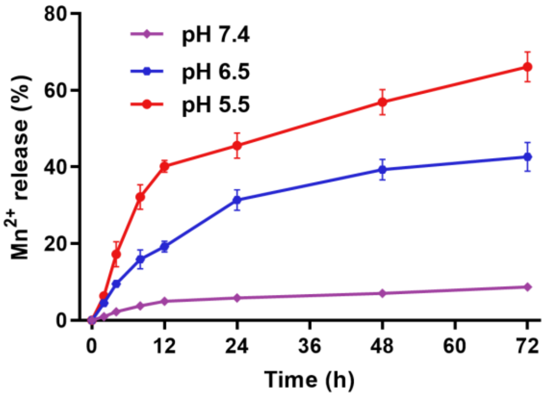

Fig. S8. *In vitro* Mn^{2+} release of MPN-Mn@E-E' in solutions at pH 7.4, 6.5, and 5.5 at 37 °C over time.

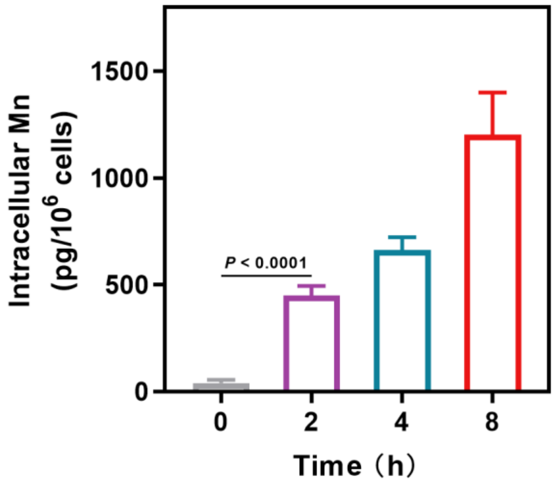

Fig. S9. Intracellular Mn content in B16F10 cells after incubation with the nanoparticles for different time periods.

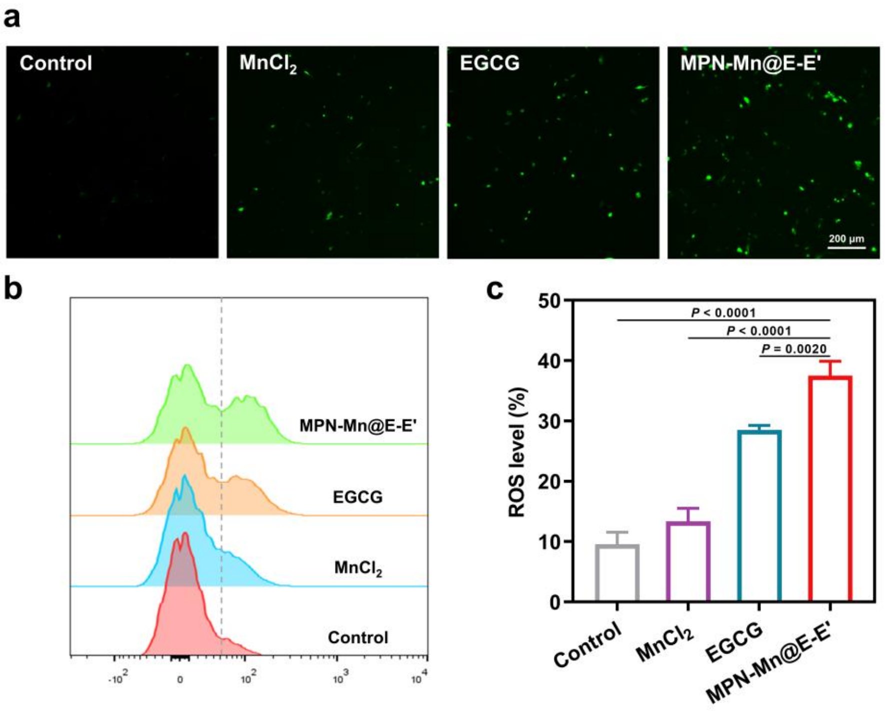

Fig. S10. Intracellular Reactive Oxygen Species (ROS) generation induced by MPN-Mn@E-E'. (a) Confocal laser scanning microscopy images of intracellular ROS levels in B16F10 cells stained with DCFH-DA after different treatments. (b) Flow cytometric analysis and (c) corresponding quantitative results of intracellular ROS levels.

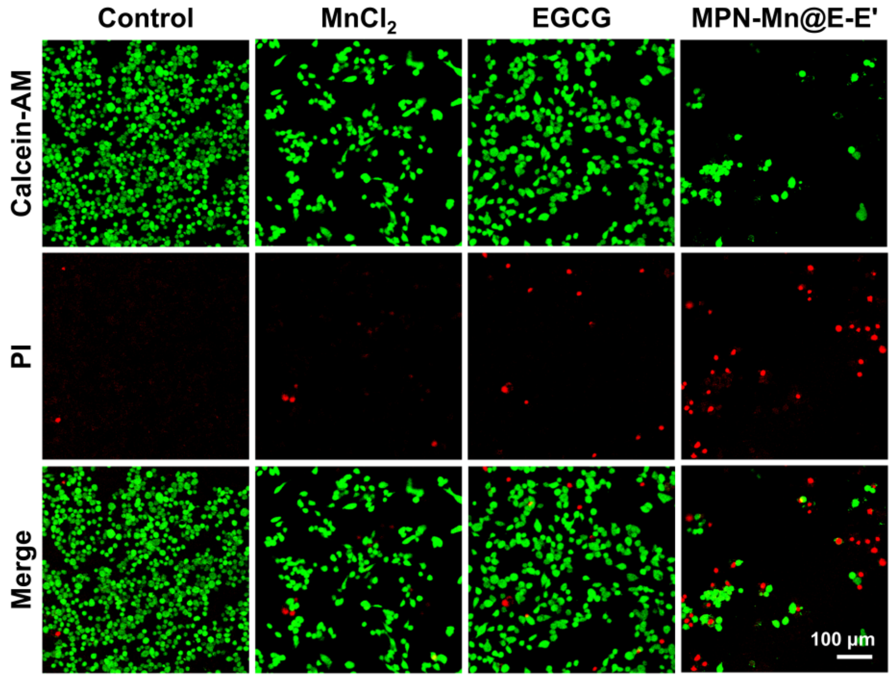

Fig. S11. Cytotoxicity evaluation by live/dead staining. Representative fluorescence images of calcein acetoxymethyl (AM)/propidium iodide (PI)-stained B16F10 cells after various treatments for 24 h.

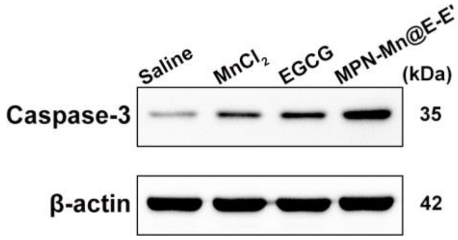

Fig. S12. Western blot analysis of caspase-3 expression in B16F10 cells under various treatments.

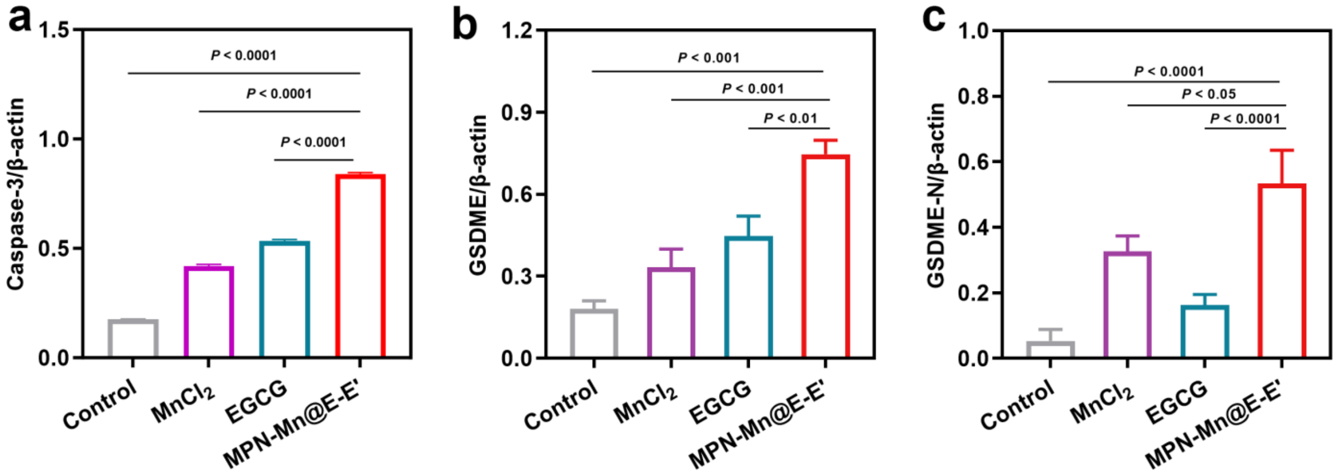

Fig. S13. Relative protein levels of (a) caspase-3, (b) GSDME, and (c) GSDME-N.

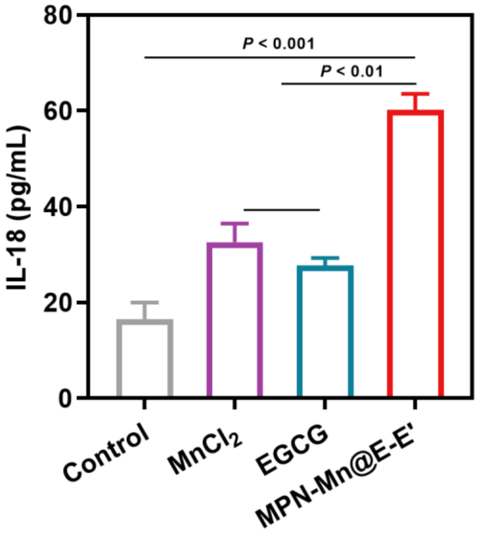

Fig. S14. Enzyme-linked immunosorbent assay to determine the levels of IL-18 in the culture supernatants of B16F10 cells.

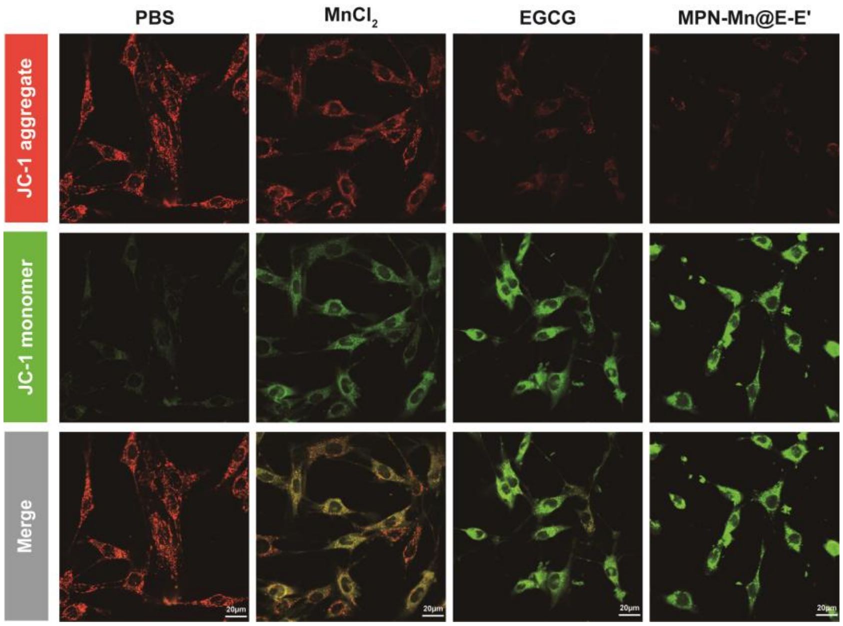

Fig. S15. CLSM was performed to observe the change of mitochondrial membrane potential. Red: JC-1 aggregate, Green: JC-1 monomer.

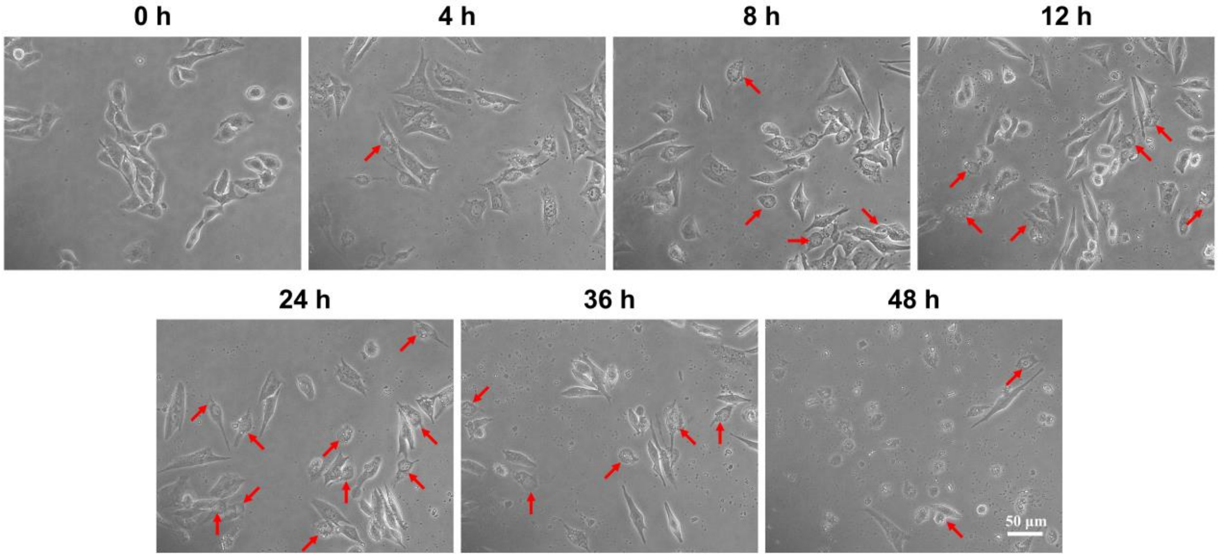

Fig. S16. Time-dependent morphological changes of tumor cells. Representative optical microscopy images of B16F10 cells at different time points following MPN-Mn@E-E' treatment.

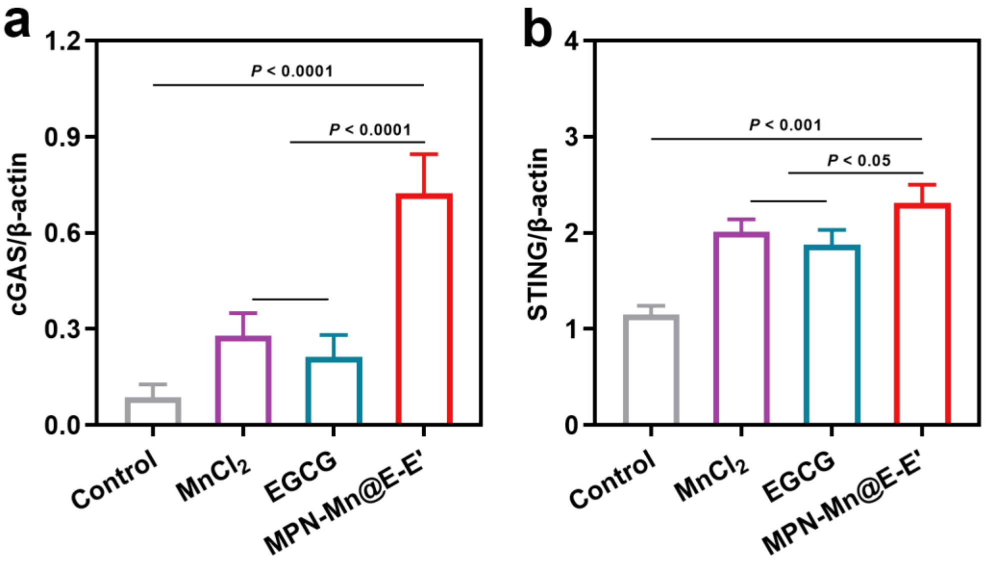

Fig. S17. Relative protein levels of (a) cGAS and (b) STING.

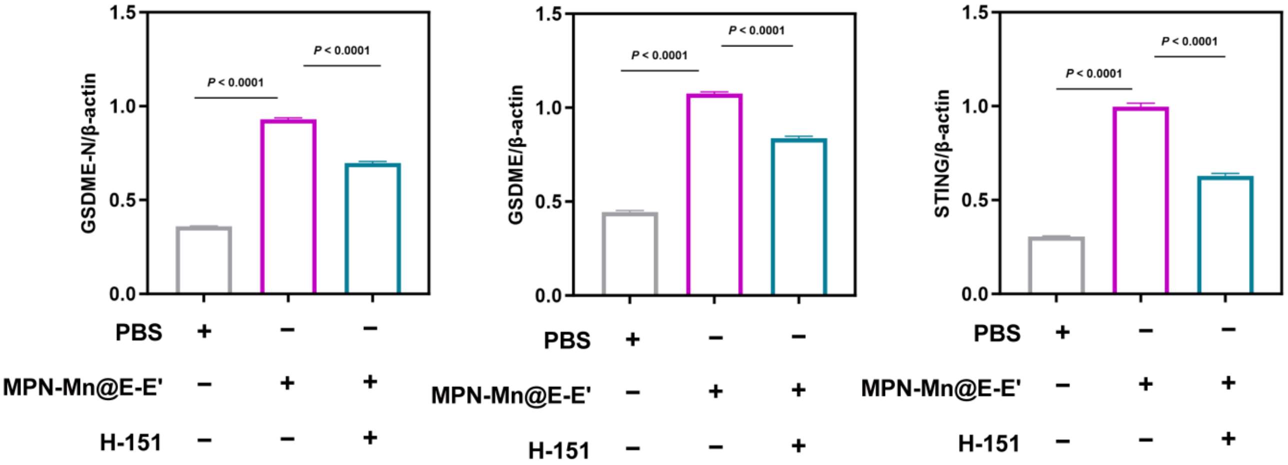

Fig. S18. Quantitative analysis of the expression levels of related proteins following the combined treatment with MPN-Mn@E-E' and the STING inhibitor H-151.

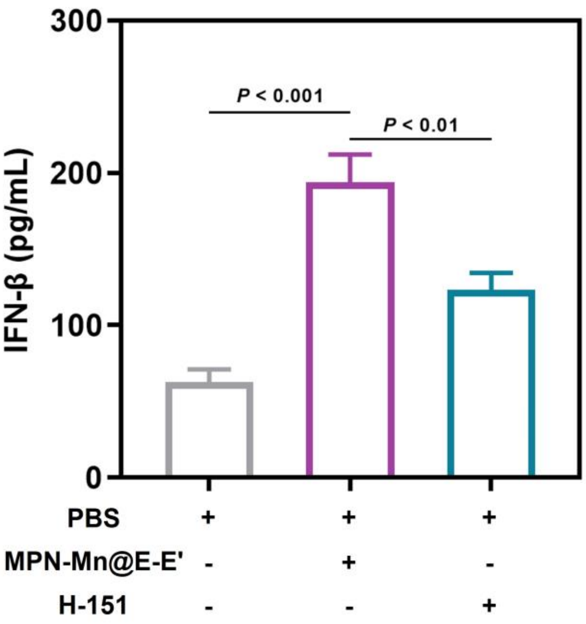

Fig. S19. Effects of the combined treatment with MPN-Mn@E-E' and the STING inhibitor H-151 on the secretion of IFN-β.

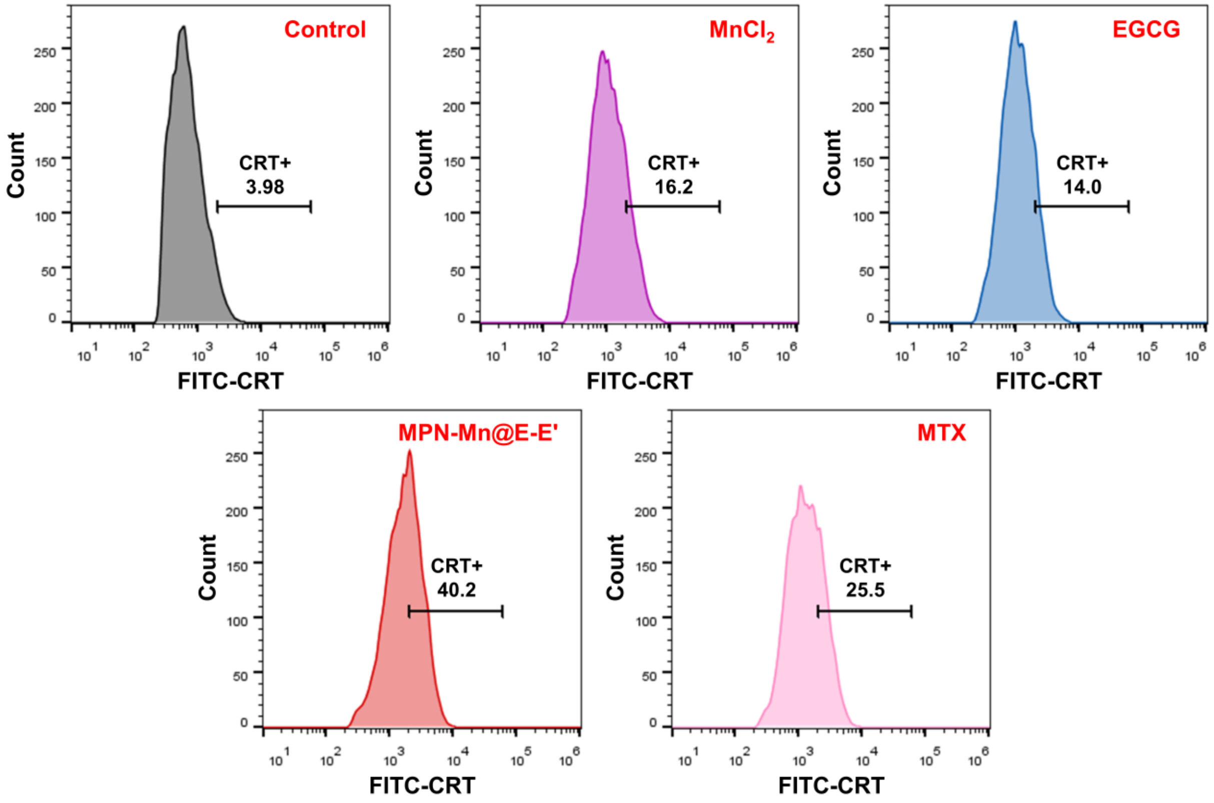

Fig. S20. Flow cytometric analysis of calreticulin (CRT) exposure. Representative flow cytometry plots of cell-surface CRT expression in B16F10 cells after different treatments.

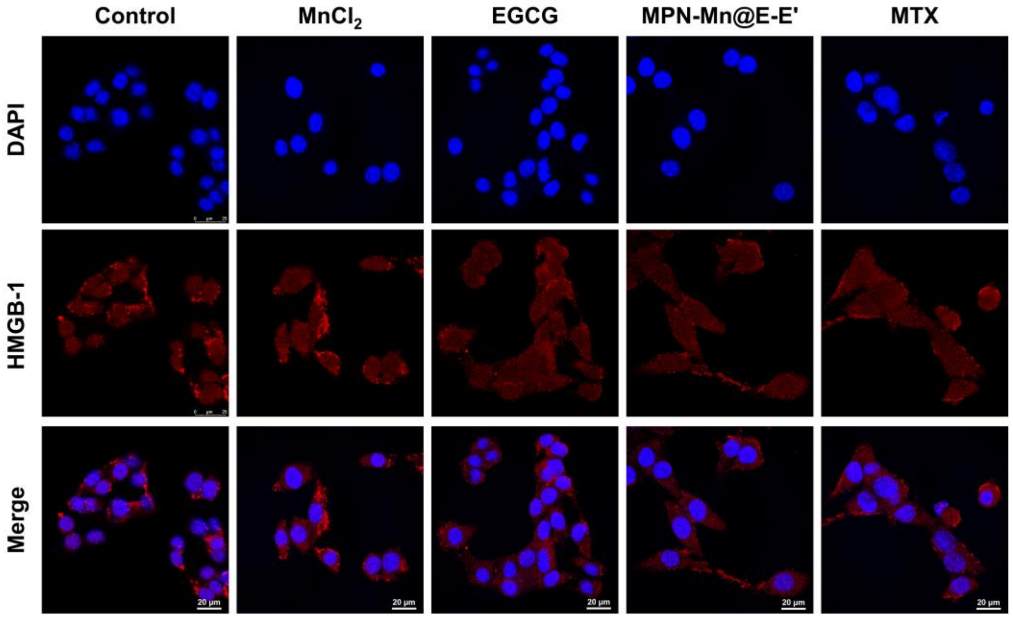

Fig. S21. High-mobility group box 1 (HMGB-1) release during immunogenic cell death. Confocal laser scanning microscopy images showing HMGB-1 release from B16F10 cells following various treatments.

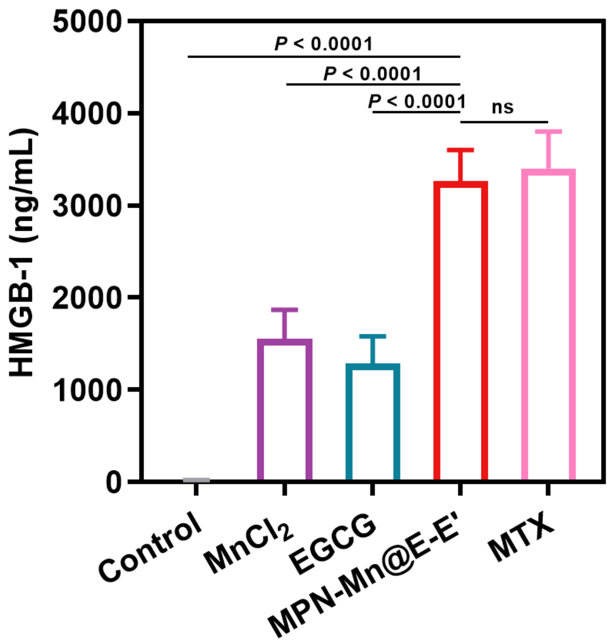

Fig. S22. Detection of HMGB-1 release from B16F10 cells following various treatments using an enzyme-linked immunosorbent assay (ELISA) kit.

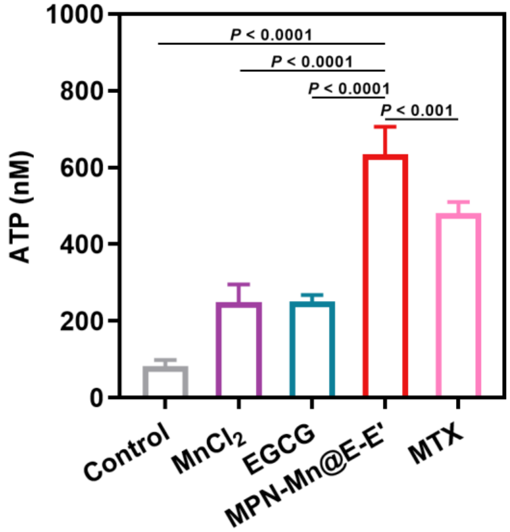

Fig. S23. Detection of ATP release from B16F10 cells following various treatments using an ELISA kit.

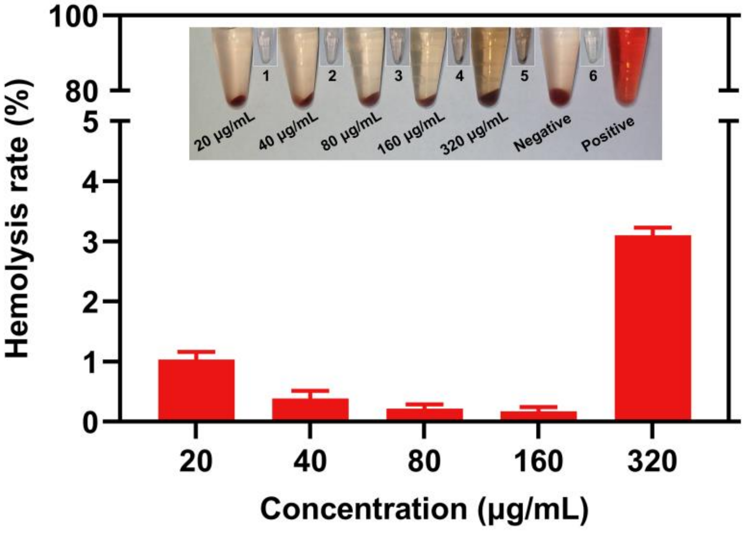

Fig. S24. Hemolysis rate and hemolysis photograph of MPN-Mn@E-E'.

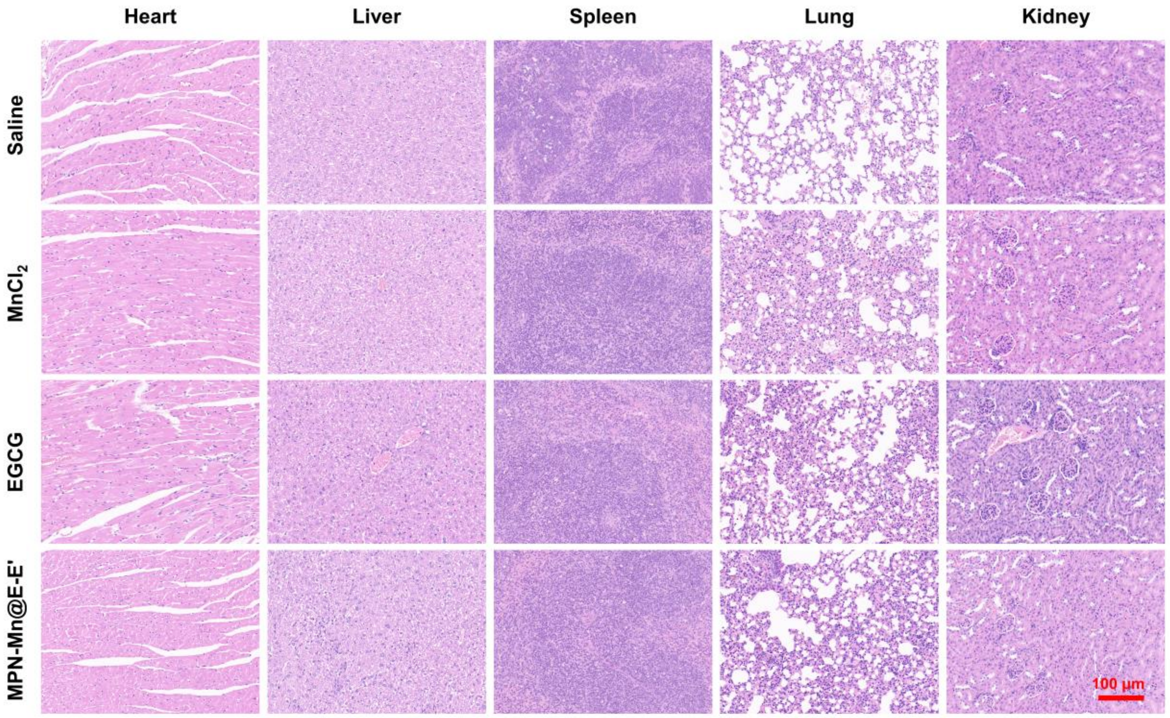

Fig. S25. Histological biosafety evaluation. Hematoxylin and eosin (H&E) staining of major organs (heart, liver, spleen, lung, and kidney) harvested from BALB/c mice treated with saline, $MnCl_2$, EGCG, and MPN-Mn@E-E'.

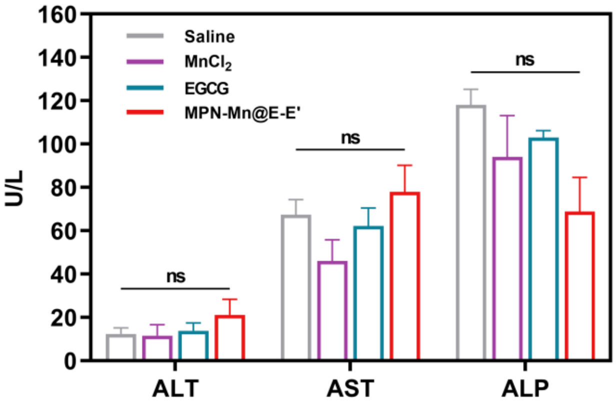

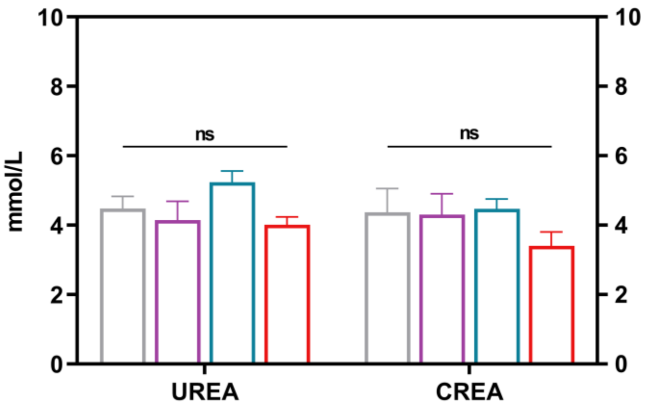

Fig. S26. Serum biochemical analysis. Serum levels of liver and kidney function markers, including alanine aminotransferase (ALT), aspartate aminotransferase (AST), alkaline phosphatase (ALP), urea (UREA), and creatinine (CREA), following different treatments.

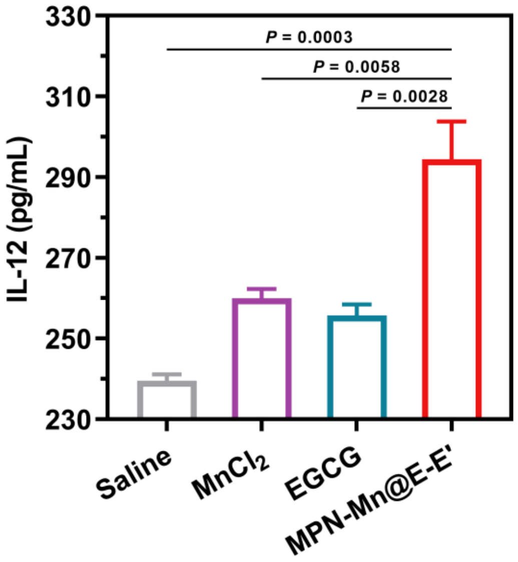

Fig. S27. Inflammatory cytokine secretion *in vivo*. Serum levels of interleukin-12 (IL-12) measured by ELISA kits in mice subjected to different treatments.

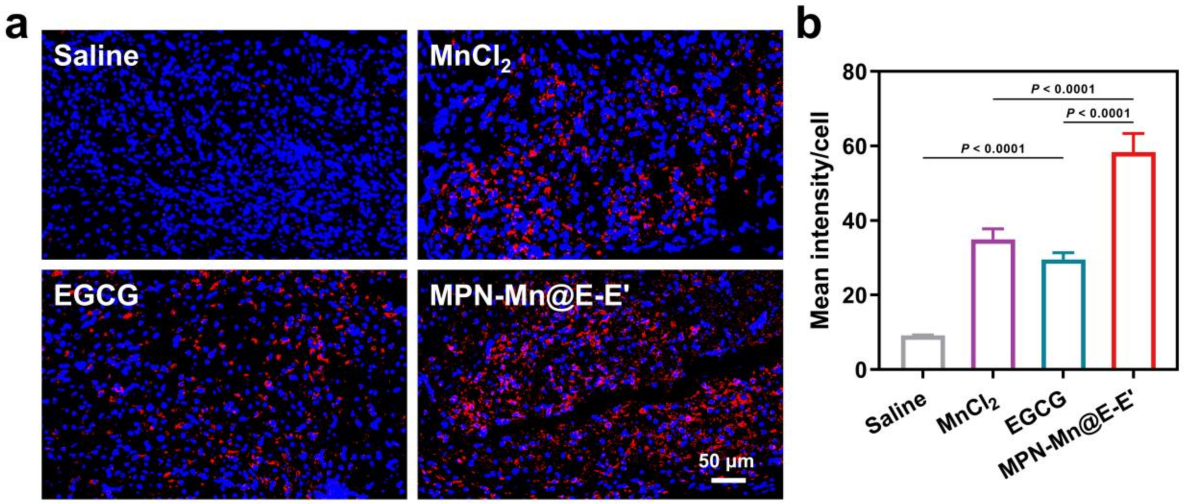

Fig. S28. *In vivo* CRT exposure. (a) Immunofluorescence staining of CRT in tumor sections and (b) semi-quantitative analysis of CRT^+ tumor cells.

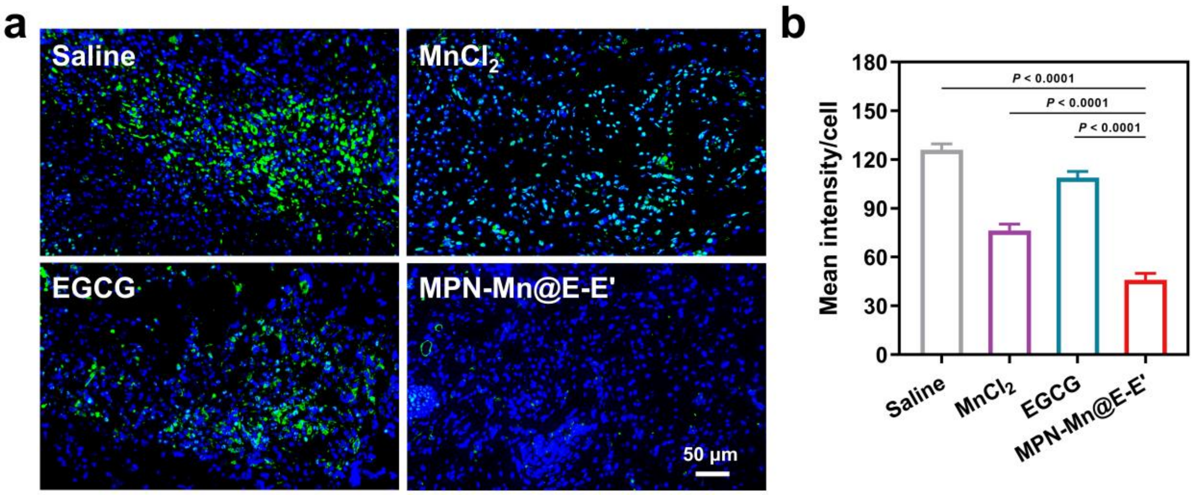

Fig. S29. *In vivo* HMGB-1 release. (a) Immunofluorescence staining of HMGB-1 in tumor sections and (b) semi-quantitative analysis of HMGB1-positive tumor cells.

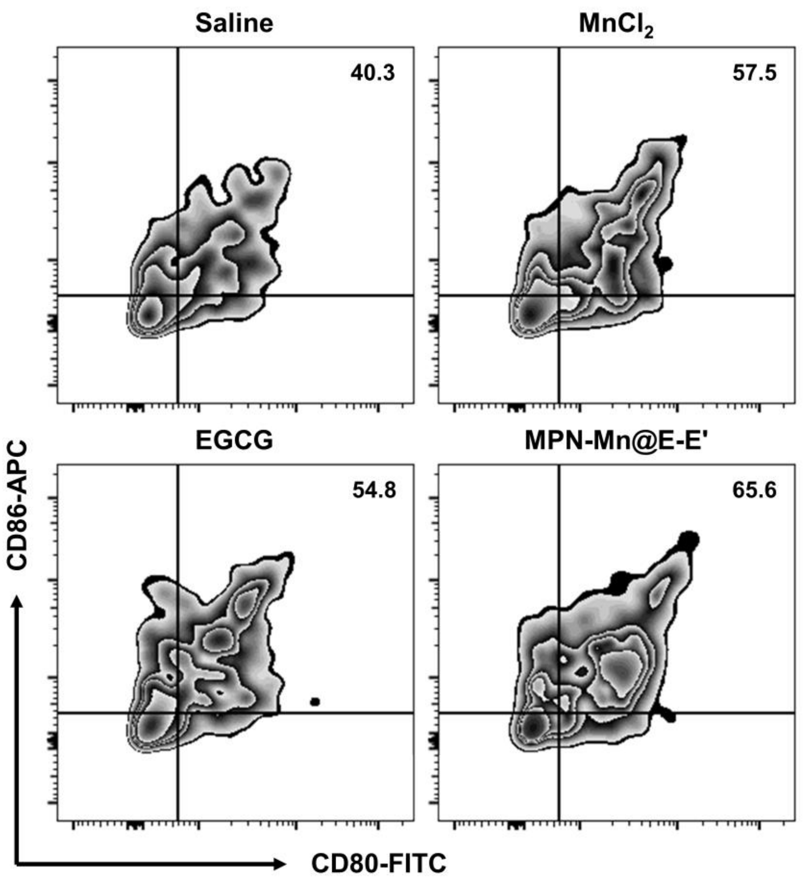

Fig. S30. Dendritic cells maturation *in vivo*. Representative flow cytometry plots of mature dendritic cells ($CD11c^{+}CD80^{+}CD86^{+}$) in the spleens of B16F10 tumor-bearing BALB/c mice.

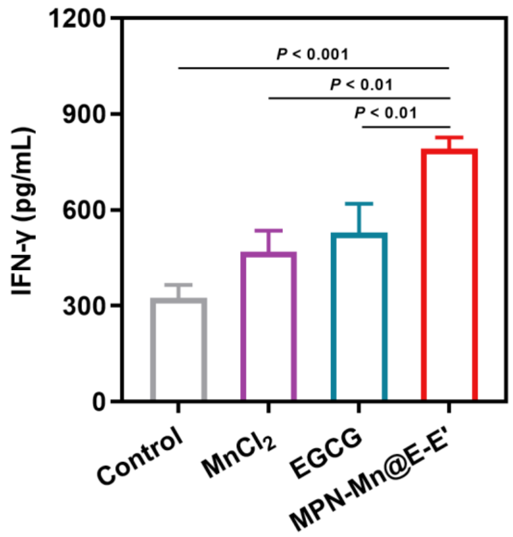

Fig. S31. Serum levels of interferon-γ (IFN-γ) measured by ELISA kits in mice subjected to different treatments.

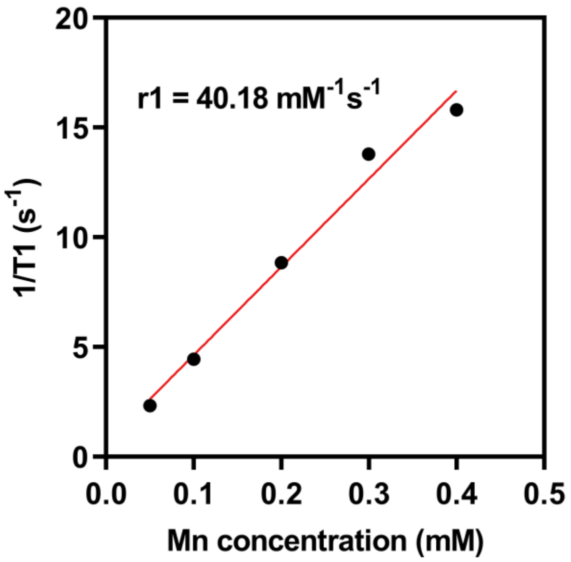

Fig. S32. Longitudinal relaxivity of MPN-Mn@E-E'. Linear correlation between T_1 relaxation rate ($1/T_1$) and Mn concentration in MPN-Mn@E-E'.

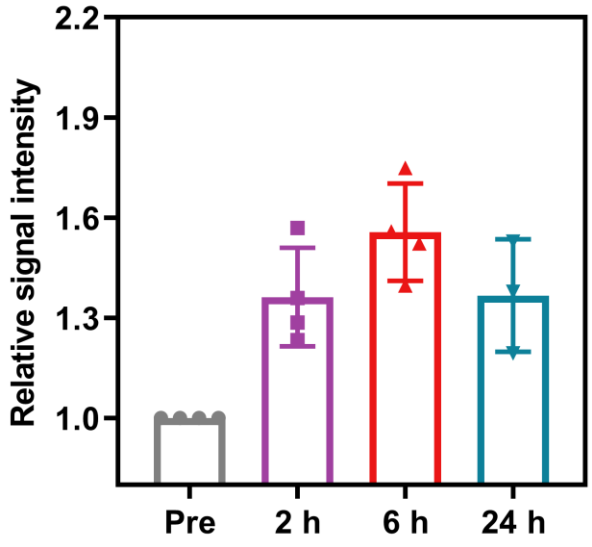

Fig. S33. Time-dependent magnetic resonance imaging (MRI) signal enhancement. Relative T_1-weighted MRI signal intensity at tumor sites at different time points after intravenous administration of MPN-Mn@E-E'.

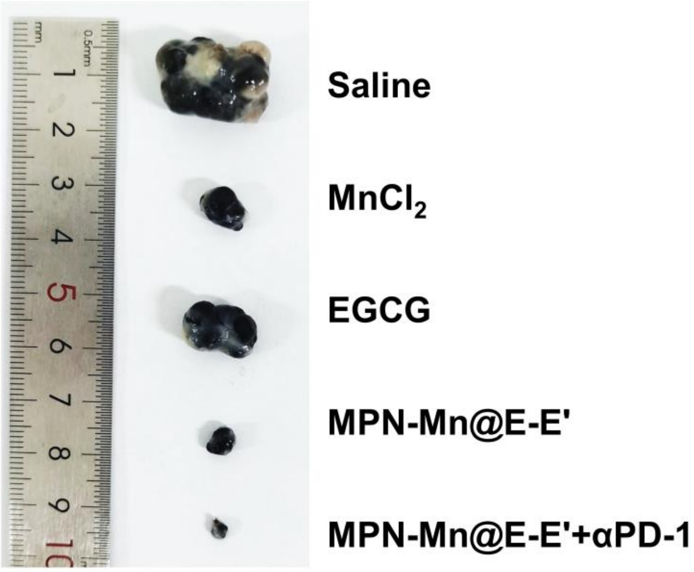

Fig. S34. Representative tumor photographs of antitumor effeciency *in vivo*. Photographs of excised tumors collected from mice in each treatment group at the end of the study.

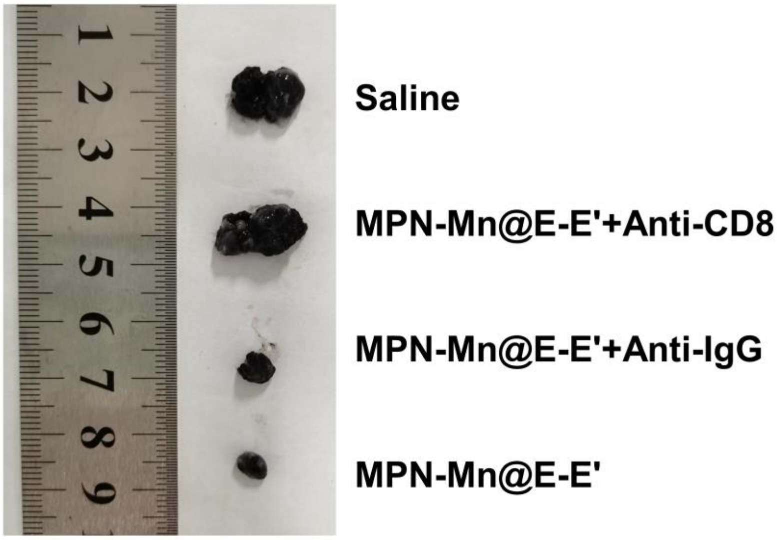

Fig. S35. Representative tumor photographs in $CD8^+$ T cell depletion study. Photographs of excised tumors from different treatment groups.
